# The killifish visual system as an in vivo model to study brain aging and rejuvenation

**DOI:** 10.1038/s41514-021-00077-4

**Published:** 2021-08-17

**Authors:** Sophie Vanhunsel, Steven Bergmans, An Beckers, Isabelle Etienne, Jolien Van houcke, Eve Seuntjens, Lut Arckens, Lies De Groef, Lieve Moons

**Affiliations:** 1 Neural Circuit Development and Regeneration Research Group, Animal Physiology and Neurobiology Section, Department of Biology, KU Leuven, Leuven, Belgium; 2 Oxurion NV, Heverlee, Belgium; 3 Neuroplasticity and Neuroproteomics Research Group, Animal Physiology and Neurobiology Section, Department of Biology, KU Leuven, Leuven, Belgium; 4 Developmental Neurobiology Research Group, Animal Physiology and Neurobiology Section, Department of Biology, KU Leuven, Leuven, Belgium; 5 Leuven Brain Institute, Leuven, Belgium

**Keywords:** Visual system, Ageing

## Abstract

Worldwide, people are getting older, and this prolonged lifespan unfortunately also results in an increased prevalence of age-related neurodegenerative diseases, contributing to a diminished life quality of elderly. Age-associated neuropathies typically include diseases leading to dementia (Alzheimer’s and Parkinson’s disease), as well as eye diseases such as glaucoma and age-related macular degeneration. Despite many research attempts aiming to unravel aging processes and their involvement in neurodegeneration and functional decline, achieving healthy brain aging remains a challenge. The African turquoise killifish (*Nothobranchius furzeri*) is the shortest-lived reported vertebrate that can be bred in captivity and displays many of the aging hallmarks that have been described for human aging, which makes it a very promising biogerontology model. As vision decline is an important hallmark of aging as well as a manifestation of many neurodegenerative diseases, we performed a comprehensive characterization of this fish’s aging visual system. Our work reveals several aging hallmarks in the killifish retina and brain that eventually result in a diminished visual performance. Moreover, we found evidence for the occurrence of neurodegenerative events in the old killifish retina. Altogether, we introduce the visual system of the fast-aging killifish as a valuable model to understand the cellular and molecular mechanisms underlying aging in the vertebrate central nervous system. These findings put forward the killifish for target validation as well as drug discovery for rejuvenating or neuroprotective therapies ensuring healthy aging.

## Introduction

Improved living conditions and advances in medicine have led to the rise in longevity that is seen today. Human populations are becoming older allover western civilizations and by 2050, one in six people will be 65 years or older. However, this observed prolonged lifespan is not necessarily accompanied by an increased health span. On the contrary, a higher prevalence of age-related central nervous system (CNS) diseases is observed in our graying society. Worldwide, 50 million people live with dementia (mostly Alzheimer’s and Parkinson’s disease) and this number doubles every 5 years^1–3^. Aging also often goes together with a decline in vision, which greatly impacts the ability to carry out everyday tasks. Indeed, for nonneurodegenerative eye conditions, including dry eye and cataract, but importantly also for neurodegenerative eye diseases, such as glaucoma and age-related macular degeneration, age is a major risk factor. Unfortunately, despite the global high incidence of neurodegenerative eye pathologies, treatments that can halt or reverse these disorders are nonexistent. Understanding the aging context seems to be crucial for finding curative therapies, as age clearly affects the integrity and functionality of the visual system, and therefore also its already limited ability to protect or repair damaged/degenerating neurons^4–6^.

Aging is defined by numerous alterations at a cellular and molecular level that eventually result in age-related deterioration of physiological functions^7^. In the brain, ten hallmarks were established: (1) mitochondrial dysfunction; (2) intracellular accumulation of oxidatively damaged proteins, nucleic acids, and lipids; (3) dysregulated energy metabolism; (4) impaired cellular waste disposal mechanisms; (5) impaired adaptive stress response signaling; (6) compromised DNA repair; (7) aberrant neuronal network activity; (8) dysregulated neuronal Ca^2+^ handling; (9) stem cell exhaustion; and (10) inflammation. Note that the manifestation of cellular senescence and telomere attrition, two hallmarks described to occur in proliferative peripheral tissues, was not convincingly proven in neurons but does occur in glial cells of the CNS^7–9^.

While many fish species, such as zebrafish and goldfish, have been exploited in biogerontology research, their relatively long lifespan complicates investigating aging in these animal models. With its relatively short lifecycle compared to other vertebrate models and by displaying several aging hallmarks that have been described for humans^7^, the African turquoise killifish (*Nothobranchius furzeri*) has been put forward as an excellent aging model to fill this gap^10–12^. Furthermore, although not yet extensively studied, its CNS has been shown to be subject to aging too, with indications for the occurrence of typical age-related hallmarks including lipofuscin accumulation, impaired protein homeostasis, mitochondrial dysfunction, reactive gliosis, and reduced adult neurogenesis, which eventually lead to impairments in locomotor, learning, and memory function^13–18^. The GRZ-AD inbred strain, in particular, is the shortest-lived of all strains, with a median lifespan of 4–6 months^19–21^. Using this model organism, age-associated changes as well as the underlying cellular and molecular processes can be investigated in depth, in order to understand and more efficiently tackle neurodegenerative events.

Over the years, the visual system has become a powerful tool to investigate the brain as a whole, both in mammals and in fish^22^. The retina is an integral part of the CNS, and disease processes occurring in the retina have shown to be an indicator of similar processes occurring elsewhere in the CNS, and vice versa. Indeed, retinal manifestations of age-related degenerative diseases, such as Alzheimer’s and Parkinson’s disease, have been documented^23–26^ and, as such, the eye is often named “a window to the brain.” Yet, the aging processes of the retinotectal system of the killifish, comprising of the retina, optic nerve, optic tract, and optic tectum, has not been characterized. Therefore, in this paper, we rigorously analyzed several age-associated processes in the retina and optic tectum of four different age groups: young adult, middle-aged, old, and very old fish. Our results reveal indications for oxidative stress, cellular senescence, reactive gliosis, inflammaging, declined neurogenic potential, and neurodegeneration in the aged killifish visual system, all together leading to age-related vision impairment. Importantly, the occurrence of these aging hallmarks and the functional decline in the retinotectal system demonstrates its value to study the cellular and molecular mechanisms underpinning brain aging, and could help elucidating physiological versus pathological aging. All in all, we propose the killifish visual system as a model to not only investigate aging processes as such, but also for the identification of new strategies for regenerative medicine, CNS rejuvenation, and healthy aging.

## Results

### Killifish growth rate decreases with age

**Fig. 1:**
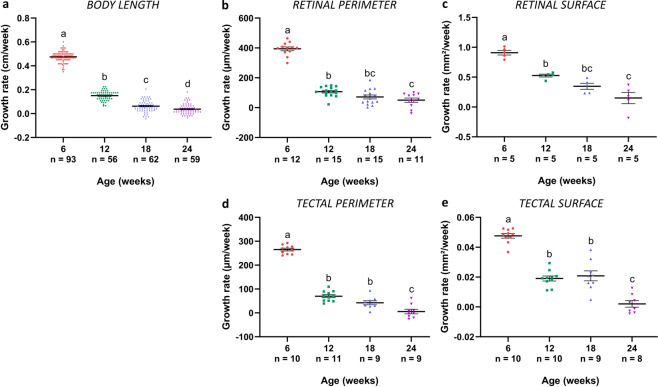
Changes in growth rate in the GRZ-AD *Nothobranchius furzeri*. **a** Growth rate of body length in 6-week-, 12-week-, 18-week-, and 24-week-old fish significantly decreases in all age groups. *n* ≥ 56. **b** Growth rate of the retinal perimeter, analyzed on mid-sagittal cryosections, significantly declines between 6 weeks and 12 weeks. Thereafter, growth rate does not reduce any further. *n* ≥ 11. **c** Also the growth rate of the retinal surface, determined on wholemounts, shows a significant decrease between the age of 6 weeks and 12 weeks. *n* = 5. **d** Growth rate of the tectal perimeter, analyzed on coronal cryosections of the central optic tectum, declines upon aging, with a significant reduction between the age of 6 weeks and 12 weeks, and between 18 weeks and 24 weeks. *n* ≥ 9. **e** Similar to the tectal perimeter, assessing growth rate of the tectal surface demonstrates a significant drop in growth rate between 6 weeks and 12 weeks, and 18 weeks and 24 weeks of age. *n* ≥ 8. All data are depicted as mean ± standard error of mean, statistical significance between different age groups is indicated using different letters.

In contrast to mammals, fish keep on growing throughout their adult life^27^. To quantify this growth and evaluate whether the growth rate of GRZ-AD killifish changes with age, the absolute body length, retinal perimeter, retinal surface, tectal perimeter, and tectal surface of 6-week-, 12-week-, 18-week-, and 24-week-old female fish, representing young adult, middle-aged, old, and very old fish, respectively, were measured and the growth rate of these parameters was calculated (Fig. 1a–e). All tested parameters showed a similar trajectory, i.e., growth rate decreased with advancing age, as has been described for both zebrafish and killifish body size^16,28^, and for zebrafish retina^29^. A high growth rate was observed in 6-week-old fish, with an increase of 31.6 ± 1.1% in body length, 27.2 ± 2.2% in retinal perimeter, 57.9 ± 2.6% in retinal surface, 26.4 ± 2.5% in tectal perimeter, and 40.1 ± 3.6% in tectal surface between 6 weeks and 12 weeks of age. Between 12 weeks and 18 weeks of age, body length only increased with 9.9 ± 1.0%, retinal perimeter with 14.3 ± 2.6%, retinal surface with 24.2 ± 3.5%, tectal perimeter with 12.6 ± 2.5%, and tectal surface with 31.2 ± 5.0%. Growth was even more confined in fish between 18 weeks and 24 weeks old (5.4 ± 0.8% in body length, 8.8 ± 2.5% in retinal perimeter, 8.4 ± 5.3% in retinal surface, 1.50 ± 2.5% in tectal perimeter, and 2.3 ± 2.5% in tectal surface). Altogether, our results reveal a rapid growth until the age of 12 weeks, which declines between 12 weeks and 18 weeks. After 18 weeks, killifish hardly grow.

### Molecular and cellular changes in the aged killifish visual system

During brain aging, numerous age-associated cellular, molecular, and systemic alterations, described as “hallmarks of aging”, lead to decreased motor, sensory, and cognitive function in elderly^8^. To characterize aging of the female killifish retinotectal system, we investigated the manifestation of several hallmarks in both the retina and optic tectum of the four age groups, i.e., oxidative stress, cellular senescence, reactive gliosis, inflammaging and stem cell exhaustion, as well as the functional phenotypes related to these changes.

**Fig. 2:**
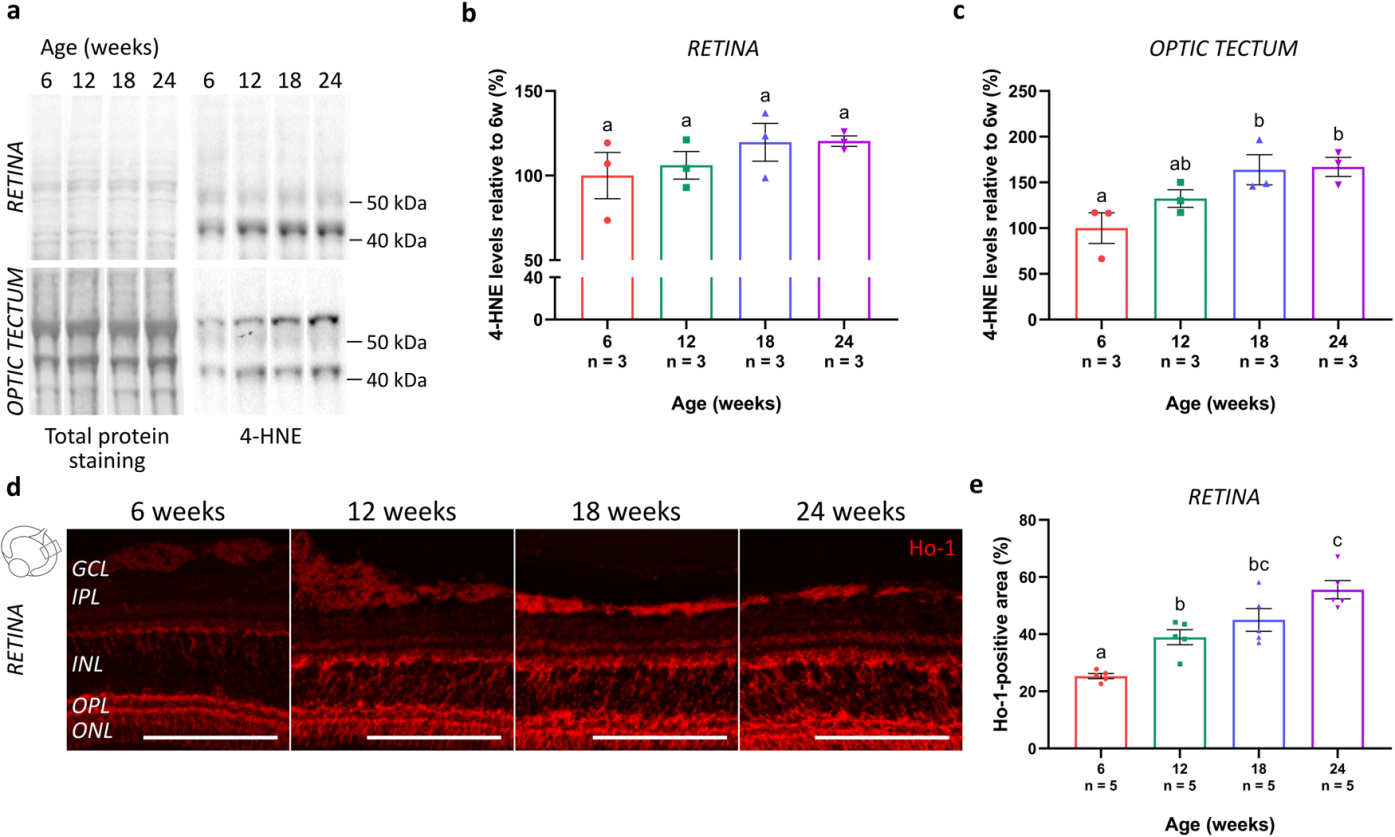
Elevated levels of oxidative stress in the aged killifish retinotectal system. **a** Representative western blot images revealing 4-HNE in the retina and optic tectum of fish of the various age groups. Quantitative analysis discloses a tendency to increased levels in the retina (**b**), and a significant age-related increase of 4-HNE levels in the optic tectum of fish of 18 weeks and 24 weeks old (**c**). All blots derive from the same experiment and were processed in parallel. Full-length blots are presented in Supplementary Fig. 3. *n* = 3. **d** Microscopic pictures of Ho-1-labeled retinal cryosections show an elevated expression of this enzyme with increasing age, especially visible in the INL. Scale bar = 100 µm. **e** Quantification of the area occupied by the Ho-1-positive signal in the retina, expressed relative to its measured surface area, reveals a significant age-associated upregulation in the killifish retina. *n* = 5. All values are shown as mean ± standard error of mean, statistical significance between different conditions is depicted using different letters. 4-HNE 4-hydroxynonenal, Ho-1 heme oxygenase 1, GCL ganglion cell layer, IPL inner plexiform layer, INL inner nuclear layer, OPL outer plexiform layer, ONL outer nuclear layer.

To assess the presence of oxidative stress in both the aged retina and optic tectum, levels of 4-hydroxynonenal (4-HNE), a byproduct of lipid peroxidation and a typical marker for oxidative damage^30–32^, were quantified using western blotting (Fig. 2a). With increasing age, 4-HNE levels tended to elevate in the retina (Fig. 2b), and significantly increased in the tectum between 6-week- and 18-week- or 24-week-old animals (Fig. 2c). In addition, immunohistochemistry for heme oxygenase 1 (Ho-1), a protective antioxidant of which the expression is typically induced in response to oxidative stress^33,34^, was performed on retinal cryosections (Fig. 2d). Quantification of the immunopositive area within a predefined region of the central retina revealed a significant elevation of Ho-1 expression with advancing age, with a most prominent increase in the inner nuclear layer (INL) of the retina (Fig. 2e). Altogether, these findings are indicative for increased oxidative stress in the aged female killifish visual system.

**Fig. 3:**
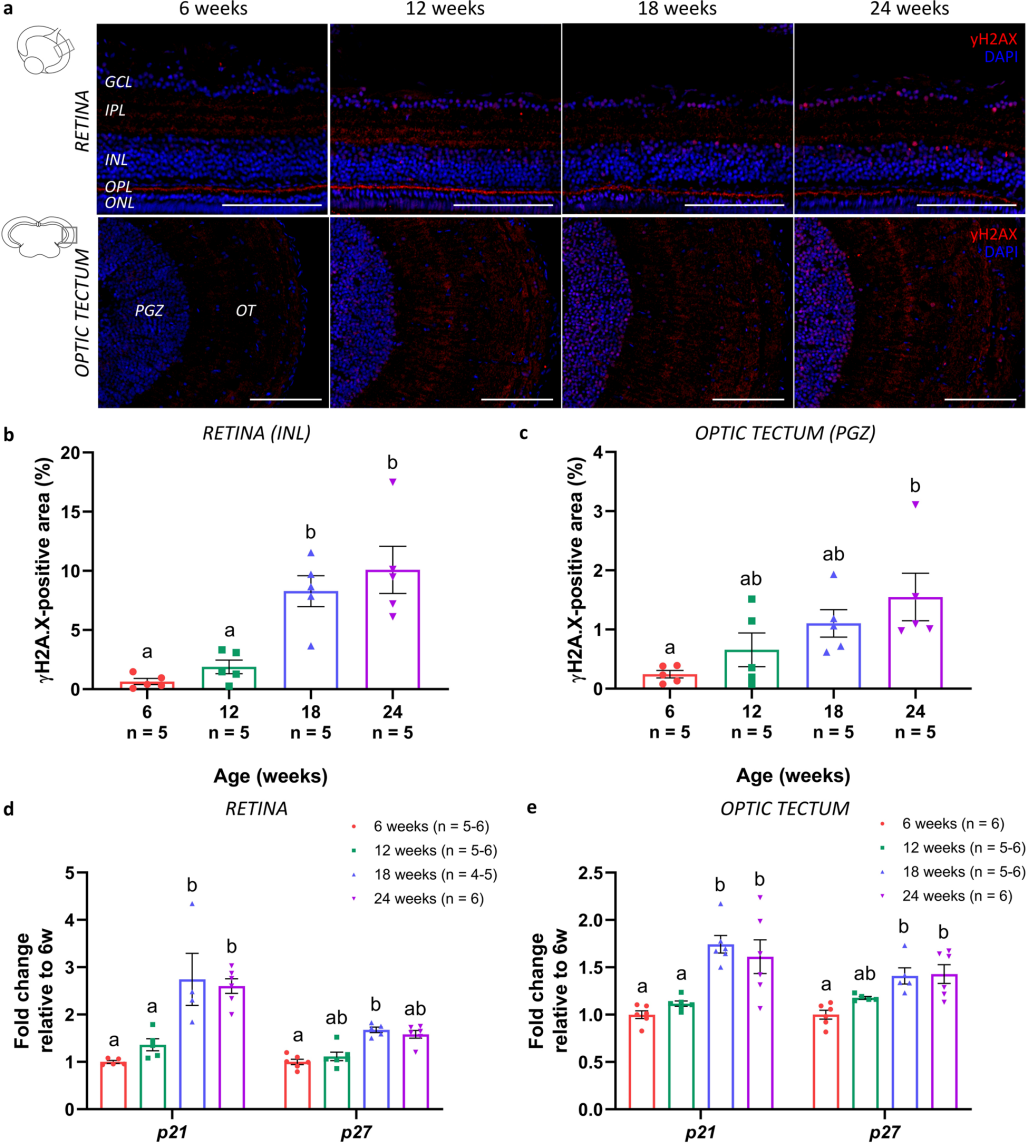
Accumulation of DNA damage and elevated levels of cell cycle regulators in the senescent killifish visual system. **a** Microscopic images of γH2AX-labeled retinal and brain cryosections show a clear accumulation of DNA damage in the nuclei, particularly visible in the GCL and INL of the retina, and the PGZ of the optic tectum. Scale bar = 100 µm. **b**, **c** Quantification of the γH2AX-immunopositive area within a predefined region of the retinal INL and tectal PGZ reveals a significant age-related increase. Values represent mean ± standard error of mean, *n* = 5. **d**, **e** Real-time quantitative PCR for cyclin-dependent kinase inhibitors *p21* and *p27*, both regulators of cell cycle progression and typical markers for cellular senescence, reveals a significant rise in gene expression levels with increasing age, both in the retina (**d**) and optic tectum (**e**). Values are mean fold change values relative to values of 6-week-old fish ± standard error of mean, *n* ≥ 4. Statistical significance between different conditions is shown using different letters. γH2AX gamma H2A histone family member X, DAPI 4′,6-diamidino-2-phenylindole, GCL ganglion cell layer, IPL inner plexiform layer, INL inner nuclear layer, OPL outer plexiform layer, ONL outer nuclear layer, PGZ periventricular gray zone, OT optic tectum.

**Fig. 4:**
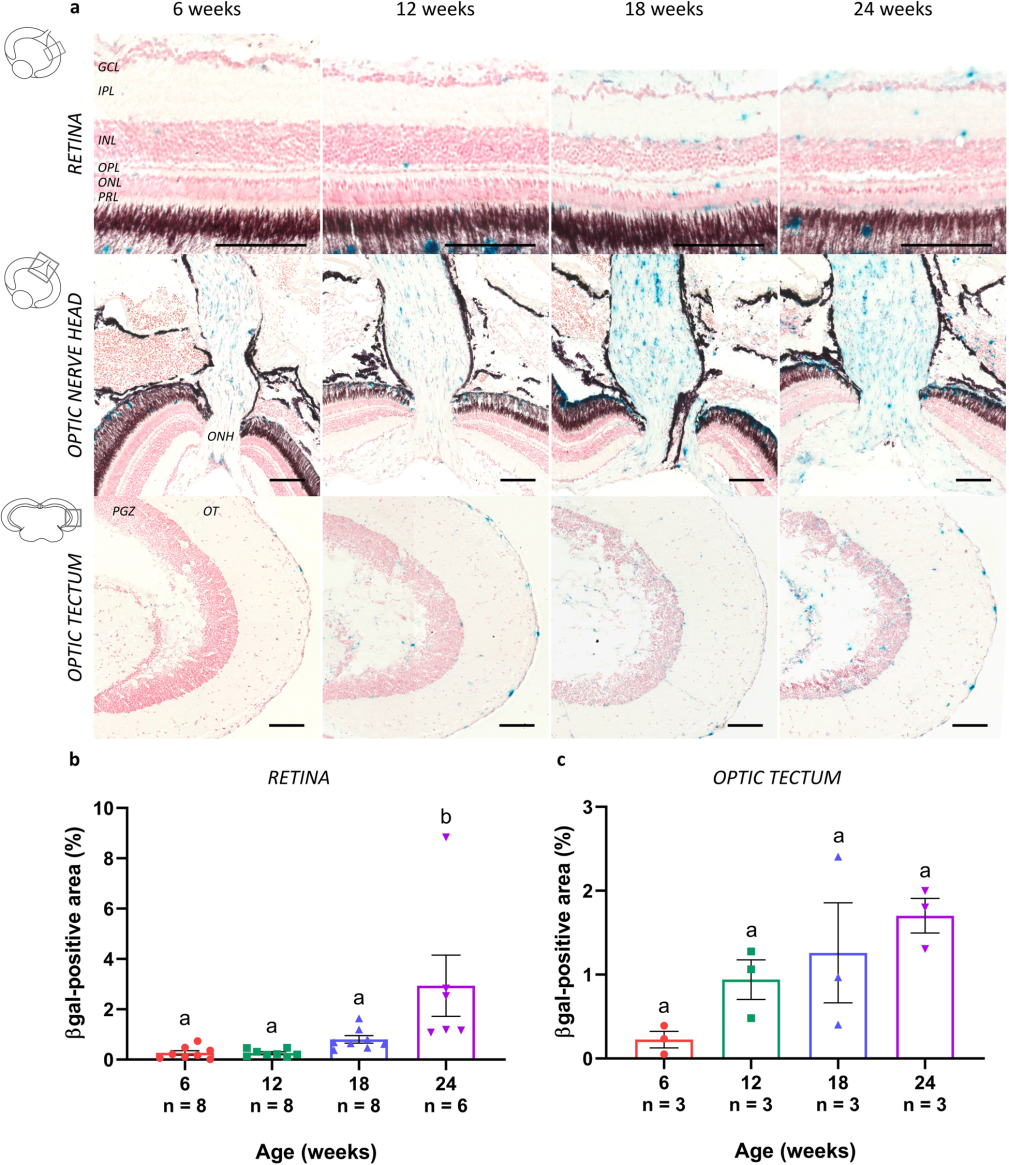
Increased SA-βgal expression in the aged killifish visual system. **a** Representative images of SA-βgal-stained sections show elevated expression levels of this lysosomal enzyme in the aged retina, optic nerve head, and brain. Whereas sections of 6-week-old fish are only scarcely labeled, increased expression levels are detected from 12 weeks onward. Note that these labeled cells are likely glial cells that are senescent. Scale bar = 100 µm. Quantification of the area occupied by SA-βgal-positive labeling demonstrates an age-associated upregulation in the neuroretina (**b**) as well as optic tectum (**c**) of the killifish. Values are shown as mean ± standard error of mean, *n* ≥ 3, statistical significance between different age groups is indicated using different letters. SA-βgal senescence-associated β-galactosidase, GCL ganglion cell layer, IPL inner plexiform layer, INL inner nuclear layer, OPL outer plexiform layer, ONL outer nuclear layer, PRL photoreceptor segment layer, ONH optic nerve head, PGZ periventricular gray zone, OT optic tectum.

The occurrence of cellular senescence in the killifish visual system was evaluated using several known senescence-associated markers. Immunohistochemistry for gamma H2A histone family member X (γH2AX; phosphorylated at the C-terminal serine-139 of the H2AX histone), an indicator of double-stranded DNA breaks that accumulate in senescent cells, on both retinal and tectal cryosections revealed a clear accumulation of DNA damage in aged killifish, especially visible in the nuclei located in the ganglion cell layer (GCL) and INL of the retina, and in the periventricular gray zone (PGZ) of the optic tectum (Fig. 3a). Quantification of the immunopositive area within a predetermined region of the retinal INL and tectal PGZ showed a significant increase in γH2AX-positive nuclei in these nuclear layers (Fig. 3b, c). Cellular senescence was further investigated using mRNA expression studies of cell cycle inhibitors *p21* and *p27*. In the retina as well as in the optic tectum, both *p21* and *p27* expression levels elevated with age and were significantly higher at 18 weeks compared to 6 weeks. After 18 weeks of age, levels did not increase any further (Fig. 3d, e). Finally, we assessed the expression of lysosomal senescence-associated β-galactosidase (SA-βgal), a putative marker of cellular senescence in peripheral proliferative tissues but also reported in the CNS^7–9^. Staining for SA-βgal on both retinal and tectal cryosections and subsequent quantitative analysis revealed a clear elevation in SA-βgal expression all over the retina, optic nerve, and in the optic tectum in killifish upon aging (Fig. 4a–c). Of note, the observed cellular staining in the optic nerve is indicative of senescence of glial cells, given that only axons and glia are present here. Together, these data suggest a rise in cellular senescence within the female killifish visual system with advancing age, especially manifesting between the age of 12 weeks and 18 weeks.

**Fig. 5:**
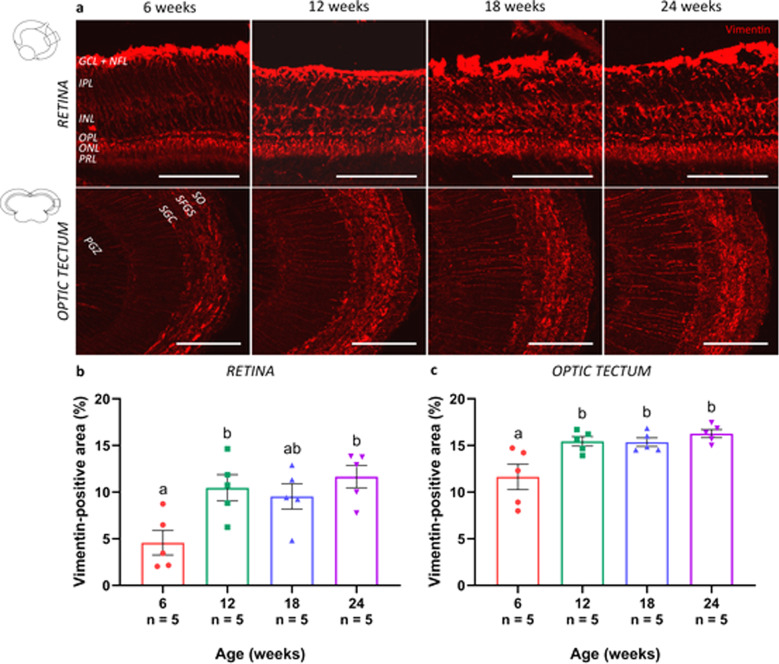
Reactive gliosis in the visual system of the aged killifish. **a** Microscopic pictures of vimentin-labeled sections of the retina (sagittal cryosections) and optic tectum (coronal cryosections) reveal a slightly upregulated expression of vimentin in 12-week-old fish when compared to young adult killifish, and a dramatic increase in fish from 18 weeks and 24 weeks old. Scale bar = 100 µm. **b, c** Quantification of the vimentin-immunopositive area in the retina and the optic tectum reveals a significantly upregulated expression of vimentin in both the retina and tectum of 12-week-, 18-week-, and 24-week-old fish as compared to young adult killifish. Values represent mean ± standard error of mean, *n* = 5, statistical significance between different age groups is depicted using different letters. GCL ganglion cell layer, NFL nerve fiber layer, IPL inner plexiform layer, INL inner nuclear layer, OPL outer plexiform layer, ONL outer nuclear layer, PRL photoreceptor segment layer, SO *stratum opticum*, SFGS *stratum fibrosum et griseum superficiale*, SGC *stratum griseum centrale*, PGZ periventricular gray zone.

In mammals, astroglial cells are known to morphologically and functionally alter with age, with an upregulation of intermediate filament proteins in Müller glia and astrocyte-like cells located in the retina and brain, respectively^35,36^. Immunostaining for the intermediate filament vimentin, followed by quantification of the labeled area, revealed a significantly augmented expression in retinal Müller glia (Fig. 5a, b) and brain radial glia (Fig. 5a, c) of older killifish when compared to young adult ones. While the dense labeling of the Müller glia endfeet did not seem to alter, detailed microscopic analysis in the killifish retina indeed disclosed a compact network of vimentin-positive radial processes spanning the entire retina from the nerve fiber layer to the outer plexiform layer, which became more prominent in aged fish (Fig. 5a, b). In addition in the optic tectum, the radial glia appeared reactivated, with fibers running from the neuronal layer of the PGZ to the superficial layers, where diffuse processes project into the *stratum griseum centrale*, *stratum fibrosum et griseum superficiale*, and *stratum opticum*, and finally terminate as endfeet on the pia surface (for a review on astrocyte-like cells in the fish brain, see ref. ^37^) (Fig. 5a, c). All in all, our data show signs of reactive gliosis in the aged female killifish visual system, already from 12 weeks of age.

**Fig. 6:**
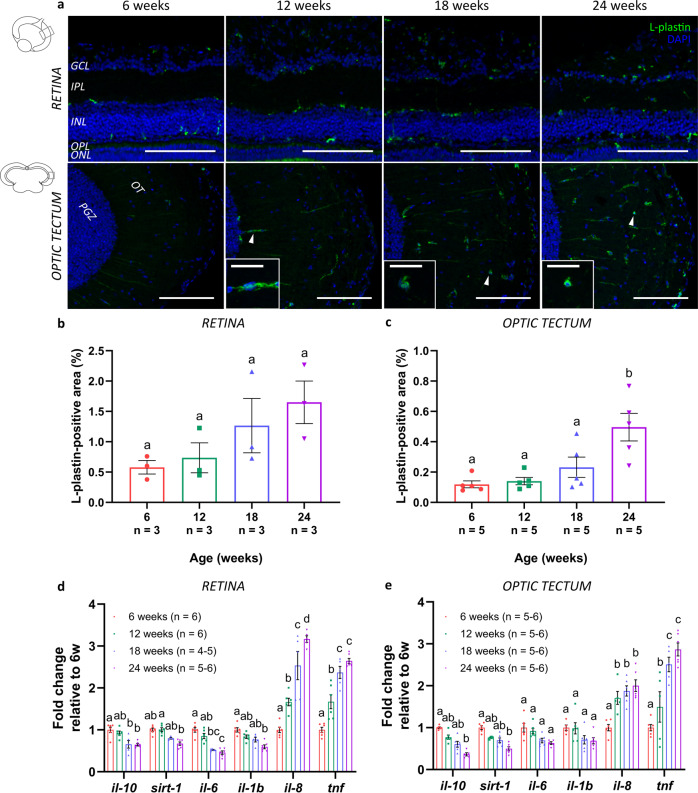
Inflammaging in the senescent killifish visual system. **a** Representative images of the retina and the optic tectum immunolabeled for the pan-leukocyte marker L-plastin illustrate a visible increase in microglial/macrophage number with increasing age. Moreover, an age-dependent change in morphology is observed in 18-week- and 24-week-old fish, in which microglia seem to transform from a ramified-like toward ameboid-like form (white arrowheads in optic tectum; magnified in boxes; scale bar = 20 µm). Scale bar = 100 µm. **b, c** Quantification of the area occupied by immunostained leukocytes over the total analyzed area demonstrates an increase in the microglial/macrophage area fraction with increasing age. Values are shown as mean ± standard error of mean, *n* ≥ 3. **d, e** Quantification of mRNA levels of inflammatory molecules, such as *il-10*, *sirt-1*, *il-6*, *il-1b*, *sirt-1*, *il-8*, and *tnf*, in both retinal and tectal lysates reveals significant changes in the expression profile in the retina as well as optic tectum with advancing age. While the expression values of *il-10*, *sirt-1*, *il-6*, and *il-1b* decline upon aging, levels of *il-8* and *tnf* increase in older fish. Values represent mean fold change values relative to values of 6-week-old fish ± standard error of mean, *n* ≥ 4. Statistical significance between different conditions is indicated using different letters. DAPI 4′,6-diamidino-2-phenylindole, GCL ganglion cell layer, NFL nerve fiber layer, IPL inner plexiform layer, INL inner nuclear layer, OPL outer plexiform layer, ONL outer nuclear layer, PRL photoreceptor segment layer, SO *stratum opticum*, SFGS *stratum fibrosum et griseum superficiale*, SGC *stratum griseum centrale*, PGZ periventricular gray zone, OT optic tectum, *il-10 interleukin 10*, *il-1b interleukin 1β*, *il-6 interleukin 6*, *sirt-1 sirtuin 1*, *il-8 interleukin 8*, *tnf tumor necrosis factor*.

As also the innate immune cell function in the vertebrate CNS is known to alter over life time, resulting in a chronic low-grade inflammatory status named inflammaging^38–41^, we next investigated the occurrence of inflammaging in the killifish visual system using immunohistochemistry for the pan-leukocyte marker L-plastin (Fig. 6a). Upon aging, the morphology of resident microglia/macrophages changed from a ramified toward a more ameboid form, particularly visible in 18-week- and 24-week-old fish (Fig. 6a; magnified boxes). Quantification of the L-plastin-immunopositive area within a predefined region in the retina and tectum revealed that, with advancing age, the area occupied by microglia/macrophages tended to increase in the retina (Fig. 6b) and was significantly elevated in the optic tectum (Fig. 6c). Next, to changes in their cell number and morphology, microglia in the aging vertebrate brain also typically display a senescence-associated secretory phenotype (SASP), with increases in proinflammatory and decreases in anti-inflammatory molecules^9,42^. Real-time quantitative polymerase chain reaction (RT-qPCR) on samples of the killifish retinotectal system disclosed an age-related reduction in mRNA levels of *interleukin 10* (*il-10*), *sirtuin 1* (*sirt-1*), *il-6*, and *il-1b* in the retina as well as tectum. On the contrary, an upregulated expression of *il-8* and *tumor necrosis factor* (*tnf*) could be observed with advancing age (Fig. 6d, e). Together, these findings indicate the occurrence of inflammaging that is accompanied by a different secretory profile in both the old retina and optic tectum of female killifish.

**Fig. 7:**
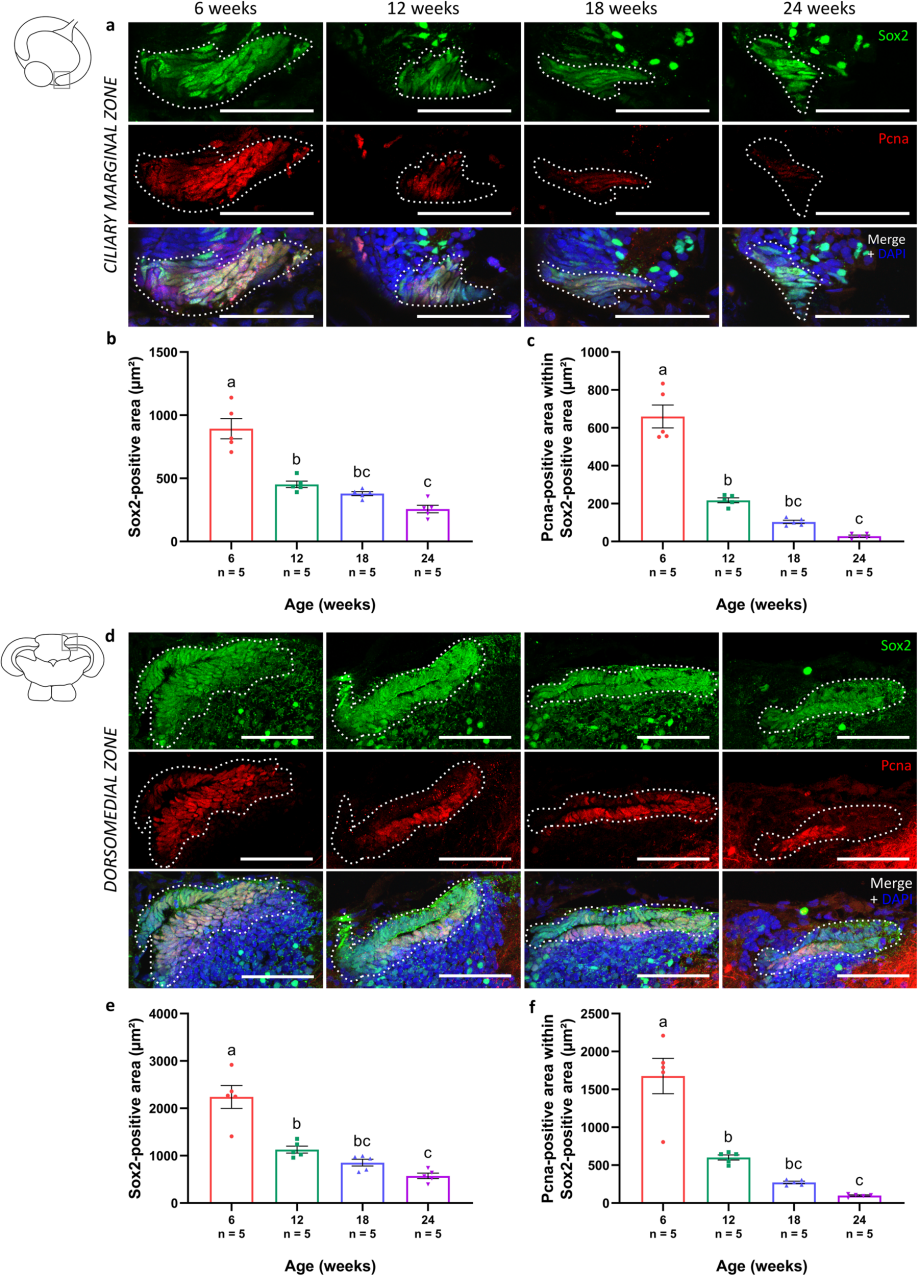
Reduced number of (proliferating) stem cells in the old killifish visual system. **a** Microscopic images of the neurogenic zones in the killifish visual system, after double labeling for Sox2 and Pcna, disclose proliferating NSPCs within the ciliary marginal zone (indicated with a white dashed line) of the young adult retina. Scale bar = 50 µm. **b, c** Quantification of the Sox2- and Pcna-immunopositive areas reveals a significant reduction in both NSPCs (Sox2-positive; dashed outline) (**b**) and proliferating NSPCs (Sox2 and Pcna double-positive) (**c**) with increasing age. **d** Proliferating NSPCs have also been identified in the dorsomedial zone of the optic tectum (depicted as a white dashed line). Scale bar = 50 µm. As a function of age, the area occupied by Sox2-positive NSPCs (dashed outline) (**e**) as well as the area covering the Sox2 and Pcna double-positive proliferating NSPCs (**f**) significantly decrease in this zone. Note that immunostaining for Sox2 is known to label a subset of amacrine cells in the mature fish retina and a subpopulation of neurons in the periventricular gray zone of the optic tectum^105–107^. All values represent mean ± standard error of mean, *n* = 5, statistical significance between different age groups is shown using different letters. DAPI 4′,6-diamidino-2-phenylindole, Sox2 sex-determining region Y-box 2, Pcna proliferating cell nuclear antigen, NSPC neural stem/progenitor cell.

Another hallmark of aging is stem cell exhaustion^43–47^. In the fish visual system, neural stem/progenitor cells (NSPCs) are situated in the ciliary marginal zone of the retina, and the dorsomedial and ventrolateral zones at the edges of the PGZ of the optic tectum. To evaluate age-associated alterations in the number of NSPCs, and whether or not they are proliferating, we labeled cycling NSPCs in these mitotic niches via double stainings for the proliferation marker proliferating cell nuclear antigen (Pcna) and the stem cell marker sex-determining region Y-box 2 (Sox2). The presence of proliferating NSPCs in the ciliary marginal zone was clearly detected in the retina of adult killifish (Fig. 7a). However, with increasing age, a decline in the area occupied by the Sox2-immunopositive NSPCs (Fig. 7b) as well as by the Sox2/Pcna double-labeled proliferating NSPCs (Fig. 7c) could be identified. NSPCs were also detected in the dorsomedial (Fig. 7d) and ventrolateral (Supplementary Fig. 1a) neurogenic zones of the adult tectum. Similar as in the retina, an age-associated decrease in both the NSPC-positive area (Fig. 7e) and its proliferative activity (Fig. 7f) was observed. All in all, these results suggest an age-related decay of the NSPC pool as well as a decline in its mitotic activity, in both retina and optic tectum.

**Fig. 8:**
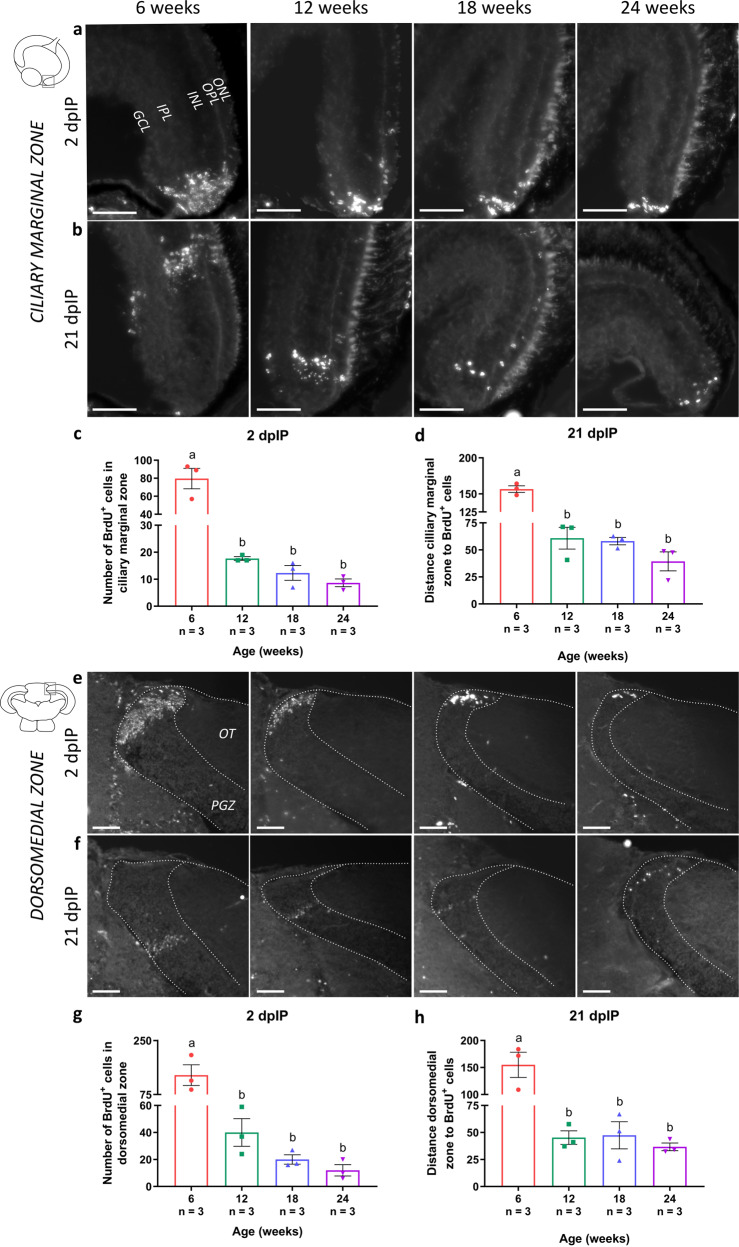
Decreased neurogenic potential in the aged killifish visual system. **a, e** Representative images of sections of all four age groups containing the neurogenic zones of the retinotectal system immunostained for BrdU. At 2 dpIP (following a 12 h pulse), BrdU-labeled cells (white dots) are clustered in the neurogenic zones of the retina and the optic tectum (depicted as a dashed line). Scale bar = 50 µm. **c, g** Quantification of the number of BrdU-positive cells in the mitotic niches at 2 dpIP reveals a clear decrease in both tissues with increasing age, and most obvious between the age of 6 weeks and 12 weeks. **b, f** Microscopic pictures of BrdU-positive cells in the retina and optic tectum after 21 days of chase demonstrate that BrdU cells are located further away from the neurogenic zones. **d, h** Measuring the distance from the neurogenic zone to the furthest BrdU-positive cell shows a dramatic decrease in distance in the older age groups. Scale bar = 50 µm. All values are mean ± standard error of mean, *n* = 3, statistical significance between different conditions is indicated using different letters. dpIP days postintraperitoneal injection, BrdU 5-bromo-2′-deoxyuridine, GCL ganglion cell layer, IPL inner plexiform layer, INL inner nuclear layer, OPL outer plexiform layer, ONL outer nuclear layer, OT optic tectum, PGZ periventricular gray zone.

To further investigate the neurogenic potential within the neurogenic zones of the killifish visual system, a 5-bromo-2′-deoxyuridine (BrdU) pulse-chase experiment with a 12-h pulse was performed, and neurogenesis was studied at 2 and 21 days after the second intraperitoneal injection (dpIP). At 2 dpIP, BrdU-positive cells were clearly visible in all mitotic zones, independent of age, indicating that new cells were generated in all age groups (Fig. 8a, e; Supplementary Fig. 1b). However, a significant decline in the number of BrdU-labeled cells could be observed with increasing age, which reinforces that the number of dividing cells in the mitotic niches declines with aging (Fig. 8c, g). Following 21 days of chase, BrdU-labeled postmitotic cells, and thus newborn cells could be visualized in all age groups. Reflecting the continuous growth of the killifish retina and brain, these cells were observed further away from the ciliary marginal zone (toward the central retina), and from the dorsomedial or ventrolateral zone (in the PGZ), where they were visualized at 2 dpIP (Fig. 8b, f; Supplementary Fig. 1b). However, in older killifish, the total number of these newly generated cells was much lower than in young adult killifish, suggesting that less cells were added as a function of age, indicative for a decreased neurogenic potential of the NSPCs (Fig. 8b, f). Furthermore, the distance from the neurogenic niche to the BrdU-positive cells at 21 dpIP was clearly shorter in the retina and tectum of older fish, which further supports that the NSPCs in these neurogenic niches divide less often (Fig. 8d, h). These findings thus show a decline in the number of (cycling) NSPCs as well as in their proliferative activity, in the aged retina as well as optic tectum.

### Functional phenotypes of the senescent killifish visual system

**Fig. 9:**
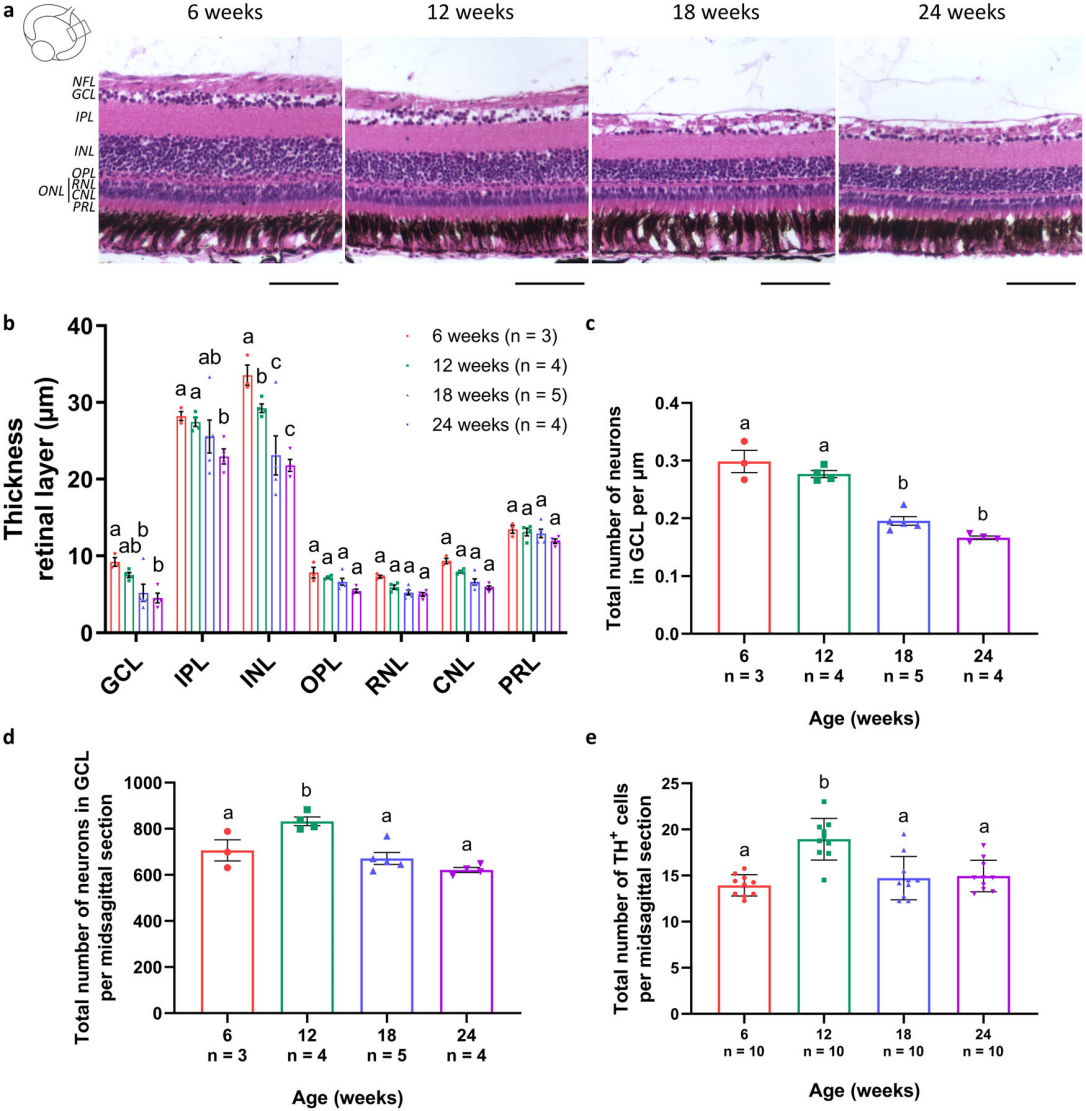
Thinning of the retinal layers upon aging. **a** Representative pictures of hematoxylin and eosin-stained retinal paraffin sections reveal a clear decrease in retinal thickness in 12-week-old fish, and this thinning further continues with increasing age. Scale bar = 100 µm. **b** Quantification of the thickness of all retinal layers on mid-sagittal paraffin sections shows a gradual thinning, with a significant decline in the GCL, IPL, and the INL. *n* ≥ 3. **c** Further quantification of the total number of neurons in the GCL per µm reveals a significant decline in cells per µm between 12 weeks and 18 weeks. *n* ≥ 3. Counting absolute numbers of neurons in the GCL (**d**) and of TH-positive dopaminergic (displaced) amacrine cells located in the GCL and INL (**e**) of the retina demonstrates an age-associated increase in cell number between 6 weeks and 12 weeks, and decrease between 12 weeks and 18 weeks of age. *n* ≥ 3 for d and *n* = 10 for e. All values are shown as mean ± standard error of mean, statistical significance between different age groups is shown using different letters. NFL nerve fiber layer, GCL ganglion cell layer, IPL inner plexiform layer, INL inner nuclear layer, OPL outer plexiform layer, ONL outer nuclear layer, RNL rod nuclear layer, CNL cone nuclear layer, PRL photoreceptor segment layer, TH tyrosine hydroxylase.

Wondering whether the observed hallmarks might lead to neurodegeneration in the killifish visual system, age-associated changes in retinal morphology were studied on hematoxylin and eosin (H&E)-stained paraffin sections (Fig. 9a). Morphometric analysis of the central retina revealed a significant thinning of the inner retina, i.e., the GCL, inner plexiform layer (IPL), and INL. GCL and INL thinning was most prominent between the age of 12 weeks and 18 weeks (30.6 ± 15.0% and 21.0 ± 8.7%, respectively), while IPL thinning was more seen between the age of 18 weeks and 24 weeks old (10.2 ± 8.4%) (Fig. 9b). Moreover, quantification of the number of neurons in the GCL demonstrated that the number of cells per µm remained rather unchanged between 6 weeks and 12 weeks, while a significant decrease was observed between 12 weeks and 18 weeks (Fig. 9c). As retinal thinning and loss of cells per µm could be due to tissue stretching, we further performed absolute cell counts in the retina. These showed a clear increase in the absolute number of GCL neurons between 6 weeks and 12 weeks, which decreased again between 12 weeks and 18 weeks of age (Fig. 9d). Specifically looking into the number of tyrosine hydroxylase (TH)-positive dopaminergic (displaced) amacrine cells, a neuronal subtype suggested to be affected by aging and in neurodegenerative diseases in mammals as well as in fish^29,48,49^, in the GCL and INL, revealed a similar increase in cell number between 6 weeks and 12 weeks of age, and decrease between 12 weeks and 18 weeks (Fig. 9e; Supplementary Fig. 2). Altogether, while these data indicate that ocular growth and expansion occur, and thereby confound interpretation of the data, at least part of the observed retinal thinning and cellular loss might reflect true neurodegenerative pathology in the senescent female killifish visual system.

**Fig. 10:**
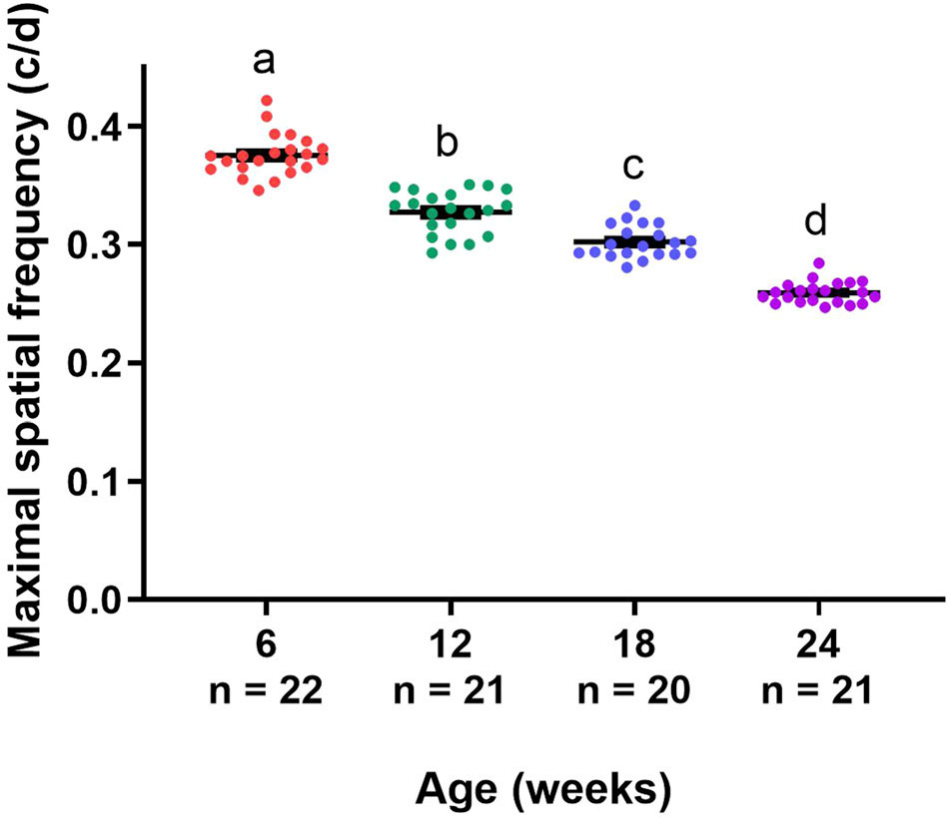
Gradual decline in visual acuity with increasing age. Evaluating visual acuity of young, middle-aged, old, and very old fish, studied by determining the maximal spatial frequency that causes an optokinetic response, discloses that all age groups retain visual acuity, yet a gradual reduction can be seen in function of age. Data are depicted as mean ± standard error of mean, *n* ≥ 20, statistical significance between different age groups is indicated using different letters.

Finally, to study whether the detected age-related alterations, potentially including neurodegenerative events, in the visual system also result in a decline in functional performance, visual acuity was evaluated in all four killifish age groups using the optokinetic response test. As shown in Fig. 10, visual acuity gradually decreased with age, which is in agreement with findings in zebrafish^50^. Aging thus clearly affects primary vision in the female killifish.

## Discussion

In 2013, López-Otín et al. identified and categorized several cellular and molecular hallmarks of aging, all contributing to the aging process and together leading to the eventual decay in performance that has been observed in aged individuals^7^. Also in the brain, many of these hallmarks have been demonstrated and shown to result in a progressive deterioration of functional abilities manifesting as declines in cognitive function, motor coordination, and sensory perception^8^. A particular functional CNS decrement that many older individuals experience is deterioration and/or loss of vision, which prevents them from performing their daily tasks. While this is partly due to nonneurodegenerative decline, vision loss in elderly is often associated with neuropathology. Indeed, alterations in the human retina during normal aging include some neuronal loss and thinning, especially in the macula, and declined visual performance, which are aggravated in age-associated neuropathologies^5,6,51–55^. Also in the visual system of the fast-aging killifish, a teleost known to recapitulate many hallmarks of human aging, we detected several age-related alterations that altogether seem to underlie a decrease in vision with age. Note that our data are obtained in female fish, and might conflict with future studies in male animals. Indeed, a large amount of evidence shows a varying prevalence for neurodegenerative diseases between sexes, with for instance Alzheimer’s disease being more prevalent in female individuals and Parkinson’s disease in males^56,57^. In addition, the pattern of neurogenesis has been demonstrated to be sexually dimorphic in the adult zebrafish forebrain, with gene expression linked with neurogenesis and cell differentiation being altered with age as well differentially expressed in the male and female brain^58,59^. Moreover, microglial sex differences have been suggested in the homeostatic (aged) mammalian brain^60^, implying that the observed inflammaging might differ in male killifish as well.

Nevertheless, our findings in female fish are a fine starting point for upcoming research as we show the occurrence of many aging hallmarks in the female killifish visual system. Accumulation of γH2AX, elevated levels of cell cycle regulators, and increased expression of SA-βgal, markers which are highly associated with augmented levels of DNA damage and proliferative arrest, are plausible indicators for the onset of cellular senescence, as already described in mammals^7,61^ as well as in teleost fish, in which cellular senescence has been shown to manifest in several tissues, including the retina and brain^15,29,62–67^. Our findings revealed an upregulation of these senescence-related markers in the aged killifish retina and tectum, suggesting that cellular senescence very likely manifests in the retinotectal system of old female killifish. Note that our assays preclude the identification of various cell types, and that we thus did not identify which cell types undergo senescence. Although CNS neurons are postmitotic and thus not presumed to become senescent, senescence-related alterations, including augmented SA-βgal signal, have been demonstrated in neurons of aged mice^9,68–70^. As a result, we cannot exclude the appearance of senescent-like neurons in the aged killifish visual system. Despite this, our observations of βgal-positive cells in the optic nerve suggest that at least glial cells show a senescent phenotype, which play an equally important role in CNS integrity and function, and are known to experience cellular senescence upon aging^9,61,71^.

One specific system that alters upon aging is the innate immune system, in which microglia, the resident immune cells of the CNS, play an essential role. With increasing age, microglia have been shown to undergo morphological changes, increase in number, and (together with neurons and other macroglial cells) alter their secretion pattern toward proinflammatory cytokines (contributing to the SASP), altogether resulting in a process termed inflammaging^9,38–40,69,72^. Similarly in the killifish, microglial number and morphology change upon aging, in the retina as well as in the optic tectum, which is in agreement with findings in the zebrafish retina and killifish brain^73–75^. Moreover, with increasing age, we demonstrate an altered secretory profile, shifting toward raised levels of proinflammatory cytokines (*il-8* and *tnf*) and a downregulation of anti-inflammatory mediators (*il-10* and sirt-1). To date, there are no reports in which inflammatory molecules contribute to inflammaging in killifish. A role for IL-8 and TNF in inflammaging has already been described in mammals, where their enhanced expression levels are typically observed in the cerebrospinal fluid and brain of aged animals^69,76–80^. IL-10, a key anti-inflammatory cytokine that suppresses mediators such as TNF and IL-8, is reported to be typically downregulated in the CNS with increasing age^81,82^, which is in agreement with our results in the killifish visual system. SIRT-1 is the most conserved member of the silent information regulator family encoding a nicotinamide adenine dinucleotide-dependent class III histone deacetylase, and has been reported to have neuroprotective as well as anti-inflammatory properties in the mammalian CNS. In line with our data, its levels, and thereby also its protective role, have been shown to progressively decline during the course of aging^83–87^. The link of Sirt-1 with aging, neuroprotection, and inflammation has also been shown in zebrafish^88,89^. Knock-out of *sirt-1* in zebrafish resulted in chronic inflammation, increased reactive oxygen species, apoptosis, and ultimately a reduced lifespan^88^. Rather unexpectedly, and although no significant differences in expression were observed in the optic tectum, levels of *il-6* were downregulated with age in the killifish retina. This contrasts mammalian models, in which IL-6 expression has been shown to increase in the old brain^68,69,80,81,90,91^. However, IL-6 is known to have both pro- and anti-inflammatory activities, so it is possible that in the killifish brain, Il-6 mainly acts as an anti-inflammatory cytokine. Of note, among the Il-6-type cytokines, leukemia inhibitory factor rather than Il-6 seems a key mediator of the inflammatory response in fish, as *lif* mRNA is upregulated in the zebrafish retina following optic nerve crush or inflammatory stimulation, while *il-6* expression remains unchanged^92,93^. Whether Il-6 might likewise not be the acting or required Il-6 type cytokine in the killifish CNS needs further investigation. Remarkably, expression of *il-1b*, recognized as a proinflammatory cytokine, diminished in the killifish visual system with increasing age. While often described to augment in the mammalian brain with advancing age^78,80,91,94,95^, not all studies show this trend of increasing *il-1b* expression^81,96^. Its function and role in the age-associated inflammatory status in killifish awaits future studies. Nevertheless, altogether, our results point toward an altered inflammatory profile in the aged killifish visual system, presented as changes in innate immune cell number, microglial morphology, and dysregulation of their secretory phenotype. In addition, our findings indicate that astrocyte-like cells in the killifish visual system are affected by age and become reactivated, as also reported in the old zebrafish retina and killifish brain^16,50^. Interestingly, in mice, the release of proinflammatory molecules such as TNF by aged microglia has been shown to activate astrocytes^97^, suggesting that inflammaging might contribute to the observed age-related reactive gliosis in killifish. These data, together with the occurrence of cellular senescence and inflammaging, indicate the significance of glia-glia and neuron–glia interactions for healthy brain aging and CNS repair in an aged setting.

Teleost fish, and thereby also their CNS, exhibit lifelong growth, which is enabled by the continued presence of numerous mitotic regions in the adult retina and brain. In the fish retina, the ciliary marginal zone allows for the constant addition of concentric rings of new neurons in the peripheral retina, and its NSPCs give rise to all retinal neurons, except for the rod photoreceptors which are generated by Müller glia-derived rod progenitors^27^. The NSPCs of the tectum are located at the peripheral margins of the tectal hemispheres and are responsible for adding concentric crescents of new neurons in the dorsomedial and ventrolateral edges of the PGZ of each lobe. Findings from our Pcna and Sox2 double immunostainings as well as a BrdU pulse-chase experiment clearly show that the NSPCs in the killifish visual system are affected by age. Indeed, in addition to a drastic decline in the number of proliferating NSPCs in the mitotic zones, our BrdU data reveal that less cells are added in aged fish, which might be indicative for a reduced survival of newborn neurons, but more importantly suggests a decreased neurogenic capacity of NSPCs. These results are in line with previously reported findings in both zebrafish and killifish, in which a decreased activity of NSPCs was proposed to lie at the basis of the decline in neurogenesis with older age^16,58,75,98^. Interestingly, as also the Sox2-positive area declines with age, our results indicate a decrease in total number of NSPCs present in these neurogenic zones. This implies that the stem cell pool diminishes upon aging, which is in agreement with findings from the subventricular zone of the lateral ventricle in aged mice^99^. What happens to these lost NSPCs remains an open question today. Whether they die or terminally differentiate when growth rate decreases is still to be determined. Notably, the fact that fish continue to grow throughout adulthood is an important factor to keep in mind when interpreting our results. Indeed, the observed reduction in cellular addition corresponds to the decline in growth rate that is detected at later ages, indicating that, at least in part, growth aspects contribute to our findings regarding stem cell exhaustion in older killifish, or more likely the other way around, i.e., reduced addition of new cells might result in a decreased growth rate (chicken–egg theory).

As already described for zebrafish, we show an age-related decline in inner retinal layer thickness^29,50,100–102^, as well as a decrease in the number of neurons in the GCL per µm. In addition, the absolute numbers of neurons in the GCL, and of dopaminergic (displaced) amacrine cells in the GCL and INL, significantly decrease at later ages. Importantly, the interplay between growth and age-associated alterations in the retina adds complexity to the interpretation of these data. Since fast growth, correlated with high neurogenic ability, is observed until the age of 12 weeks, the increase in absolute number of cells in the inner retina of 12-week-old fish compared to 6-week-old fish is likely due to cellular addition via the ciliary marginal zone of the retina and thus growth of the retina. In addition to growth by cellular addition, growth of the fish retina and optic tectum is also effectuated by tissue stretching, which results in increased distance between cells^27,29,103^ and might contribute to the observed retinal thinning and decrease in cell number per µm in the GCL. Our results indeed confirm retinal stretching between 6 weeks and 12 weeks of age, as the absolute number of neurons in the GCL increases but the number of neurons per µm remains fairly unchanged. The role of both cellular addition and retinal stretching during retinal growth in fish of different ages has been elaborately described by Van houcke et al., who reported that, at older age, growth is mainly supported by cellular addition and not tissue stretching^29^. As growth slows down in the killifish after the age of 12 weeks, just like it does after 24 months in zebrafish, we can assume a similar mechanism. A diminished neurogenic potential presumably drives the slower growth rate at later ages, but cannot account for the significant reduction in the absolute number of cells in the inner retina of 18-week- and 24-week-old fish when compared to 12-week-old fish, indicative for a loss of neurons and thus neurodegeneration at older age. Neuronal cell death was investigated via immunohistochemistry for activated-caspase-3 on retinal sections, yet no significant difference in the number of apoptotic cells was detected amongst the different age groups (data not shown). In our opinion, however, this does not exclude cell death, as neurodegeneration manifests gradually and over a long timescale, and this marker only labels cells in a specific phase of the apoptotic process, at one specific moment in time. Altogether, our findings suggest an outspoken correlation of growth and retinal changes before 12 weeks of age, which tends to decrease at later ages, thereby showing the coexistence of a decreased growth rate and neurodegenerative signs. Interestingly, neurodegeneration has also been shown in several brain areas of *Nothobranchius* species, first by Terzibasi et al., and later by Liu et al., who detected neurofibrillary degeneration in the aged optic tectum, telencephalon, brainstem, and cerebellar cortex using the Fluoro-Jade B dye^64,65,104^. More recently, an age-dependent deterioration of dopaminergic and noradrenergic neurons, and signs for α-synuclein and amyloid pathology have been reported in the killifish^48^. Our data now confirmed this dopaminergic cell loss in the aged killifish retina.

Despite the advances that have been made in biogerontology research regarding the molecular and cellular changes that occur during aging, the mechanisms of physiological aging and their interrelation with the development of neurodegenerative disorders definitely require further studies. The African turquoise killifish, which ages within a few months and experiences similar aging processes that take years in other vertebrates, provides a unique model to grasp the underlying cellular and molecular aging processes, and to identify how to tackle them. We introduce the retinotectal system of female fish as an excellent and well accessible in vivo system to understand physiological aging and age-related neuropathies. In addition, the onset of the described age-related changes can be exploited at morphological, molecular, and even functional level, and therefore used as readouts for the efficacy of supportive and/or restorative interventions, hereby positioning this fish as a useful model for drug discovery for healthy aging, brain rejuvenation, and neuroregenerative approaches.

## Methods

### Fish and husbandry

Killifish (*N. furzeri*) on a GRZ-AD background (named after Gona Re Zhou National Park in Zimbabwe where they were originally collected; later maintained by Alexander Dorn) were used and kept under standardized conditions, i.e., a 12 h/12 h light/dark cycle and water parameters consisting of a constant water temperature of 28.3 °C, conductivity of 600 µS and pH 7. Adult fish were housed in a ZebTec system (Tecniplast) with a density of four fish (three females; one male to assure female fish can spawn their eggs) per 3.5 L tank and fed twice a day with frozen red bloodworms (*Chironomidae*; Ocean Nutrition). As male and female fish differ in size and to exclude this size variation within one age group, all experiments were performed using female fish only. We used four adult age groups, i.e., young (6 weeks), middle-aged (12 weeks), old (18 weeks), and very old fish (24 weeks), which were determined based on lifespan experiments performed in our killifish facility (for details see ref. ^73^). Female fish of the GRZ-AD strain reach sexual maturity around 5 weeks of age. Fish of 6 weeks, in which no mortality was yet observed, were considered young adults. The middle-aged group of 12 weeks was determined by a 90% survival rate. Fish of 18 weeks were denoted old fish as, at this age, we observed an increased mortality and only 70% of the population was still alive. Fish of the oldest population, i.e., very old fish, had an age of 24 weeks, in which only about 50% of the population was still alive. For all experiments (except for the growth rate experiment), uniformly sized fish were used, with body lengths of 2.85 ± 0.03 cm for 6-week-old fish, 3.75 ± 0.03 cm for 12-week-old fish, 4.13 ± 0.04 cm for 18-week-old fish, and 4.35 ± 0.03 cm for 24-week-old fish. All experiments were approved by the institutional Animal Ethics Committee of the KU Leuven and strictly followed the European Communities Council Directive of 20 October 2010 (2010/63/EU).

### Growth rate determination in the killifish retinotectal system

For assessment of the growth rate of GRZ-AD killifish, body length, retinal perimeter, retinal surface, tectal perimeter, and tectal surface were determined. Body length was measured as the distance from the snout to the end of the tail fin. Retinal surface was determined on wholemounts, and retinal perimeter and tectal perimeter/surface were assessed, respectively, on central sagittal and coronal sections (see below), all using FIJI. These parameters were evaluated using female fish of all four age groups, i.e., in young adult, middle-aged, old, and very old killifish. Next, growth rate was calculated as the difference in length/perimeter/surface between two consecutive age groups, divided by six to obtain a growth rate value per week. Per age group, at minimum five fish were analyzed.

### RNA isolation and RT-qPCR

To assess the transcript levels of several age-associated genes in the killifish visual system, RT-qPCR was performed on both whole retina and optic tectum samples of young adult, middle-aged, old, and very old killifish. Briefly, fish were euthanized using 0.1% Tris-buffered tricaine, after which retinas and optic tecti were quickly dissected on ice. Per fish, the left as well as the right retina/optic tectum were pooled and digested using Tri reagent (Sigma-Aldrich). Total RNA was purified by the NucleoSpin RNA isolation kit (Macherey-Nagel, Germany). Next, oligo dT primers and Superscript III reverse transcriptase (Invitrogen) were used to reverse transcribe RNA to cDNA. All RT-qPCR reactions were carried out using SYBR Green master mix (Bio-Rad) and a CFX96 Touch Real-Time detection system (Bio-Rad). The samples were run in duplicate, with at least four independent samples per age group and an annealing temperature of 60 °C. Selection of reference genes and determination of gene expression values were performed using qBase software (Biogazelle). Primer sequences are listed in Table 1. All values are represented as expression values relative to 6-week-old values, normalized against the geometric mean of our selected reference genes.

**Table 1:** List of primer sequences used for RT-qPCR analysis.

Gene	Primer sequence (5′> 3′)
*18s ribosomal RNA*	F: ccgacattgacctcaacaa
	R: tggctgtatttgccatcc
*Elongation factor 1α*	F: actctggcattgtcgtttag
	R: agttaccagcagctttcttc
*p21*	F: cgagccctcagtactctaa
	R: gaggtttgtcggagaagaag
*p27*	F: gctcctcaatcgccatatt
	R: catacgtcgaagtggaagac
*Tumor necrosis factor*	F: caggctcacaagaggttatt
	R: ccagaggtcaatctgtcttatc
*Sirtuin 1*	F: cgagccattctaagggattt
	R: ctgaccacatcttccaaagt
*Interleukin 8*	F: acaaatcctgaccacaagtag
	R: atcgtattcaccatcatgtctc
*Interleukin 6*	F: ggaggaatttcaagggaacata
	R: cctcaggagaaccatgtaga
*Interleukin 10*	F: ccttacagcatcatgtctctc
	R: tggcagcagtcgtttatg
*Interleukin 1β*	F: ccgacagcaagaaacgaa
	R: gtggcaggacaggtataga

*RT-qPCR* real-time quantitative polymerase chain reaction, *F* forward, *R* reverse.

### BrdU labeling

For labeling of newly generated cells in the neurogenic zones of both the retina and the optic tectum, fish were injected with BrdU (Sigma-Aldrich). Killifish were briefly anesthetized in 0.03% Tris-buffered tricaine and intraperitoneally injected with BrdU solution (8 µg/g body weight). Two injections were performed with a 4-h interval. The animals were sacrificed at 2 or 21 dpIP. To visualize newborn cells, immunohistochemistry for BrdU was performed on cryosections as described below. Prior to the blocking step, DNA breaks were induced by incubating the slides in 2 N HCl at 37 °C, followed by incubation with 0.1 M borate buffer to neutralize the acid. Per experimental condition, the retina/optic tectum of at least five fish were morphologically evaluated and three of them were quantitatively analyzed. On the 2 dpIP sections, BrdU-positive NSPCs within the neurogenic zones, i.e., the ciliary marginal zone of the retina, and the dorsomedial and ventrolateral zones of the tectum, were counted. For the sections of the 21 dpIP condition, the distance from the neurogenic niche to the most distal cell of the BrdU-positive cell cluster was measured. Quantification was performed on six sections per fish, and then averaged per fish.

### Immunohistochemistry and histomorphometric analysis

For immunohistochemistry and histomorphometric analysis, killifish were euthanized by submersion in 0.1% Tris-buffered tricaine, and transcardially perfused with phosphate-buffered saline (PBS; 0.01 M; pH 7.4), followed by 4% paraformaldehyde (PFA) in PBS. Next, eyes and brains were dissected and postfixed in PFA for 1 h at room temperature (for immunolabeling) or overnight at 4 °C (for SA-βgal staining), or overnight in Bouin Hollande at 4 °C (for H&E staining). Following appropriate tissue processing, 7 µm paraffin sections or 10 µm cryosections of the retina (sagittal), and 10 µm cryosections of the brain (coronal, at the level of the optic tecti) were made. After stainings, sections were mounted with Mowiol or DePeX, and covered with a glass cover slide.

Retinal morphology was studied on H&E-stained paraffin sections. Histological pictures were taken with a microscope Leica imager at 20× magnification. For morphometric analysis of the retinal thickness, all different layers of the retina were measured on sections through the central retina at 200 µm from the optic nerve head, at the dorsal as well ventral side of each section. In addition, neurons in the GCL were counted over the entire perimeter of the retina. Using FIJI software, at least three mid-sagittal sections (max 240 µm from the central section) per fish were analyzed. All measurements were then averaged per fish. Three to five fish were studied per age group.

Expression of SA-βgal was investigated on cryosections of both eyes and brains, pretreated in PBS pH 6. Next, the slides were incubated overnight in 5 mM potassium ferrocyanide, 5 mM potassium ferricyanide, 2 mM MgCl_2_, and 1 mg/ml X-Gal (Panreac Applichem) in PBS pH 6 at 37 °C. Using a Zeiss Imager Z1 microscope, pictures at 20× magnification were made. Per age group, at least three fish were quantified by determining the area occupied by SA-βgal signal over an entire retinal section (covering all neuroretinal layers) or tectal section (including both the PGZ and optic tectum) via automated thresholding using FIJI (RenyiEntropy), which was then put relative to the total measured surface area.

Immunolabelings were performed on cryosections. The following primary antibodies were used: rat anti-BrdU (1:400; Abcam), rabbit anti-Ho-1 (1:100; Enzo Life Sciences), rabbit anti-L-plastin (1:200; Genetex), mouse anti-Pcna (1:500; Abcam), rabbit anti-Sox2 (1:1000; Sigma-Aldrich), rabbit anti-TH (1:400; Merck-Millipore), mouse anti-vimentin (1:400; Sigma-Aldrich), and rabbit anti-γH2AX (1:200; Genetex). Detailed information of the primary antibodies used is listed in Table 2. Alexa-conjugated secondary antibodies or a horseradish peroxidase-labeled antibody (Dako) in combination with the Tyramide Signal Amplification technology (TSA^TM^; PerkinElmer) were used for detection. All immunostainings were visualized using an Olympus FV1000 confocal microscope at 20× or 60× magnification. TH-positive cells were counted over the entire retina on at least four mid-sagittal sections, and then averaged per fish. Ten fish were studied per age group. Stainings for Ho-1, L-plastin, Pcna/Sox2, vimentin, and γH2AX were quantified using FIJI by determining the immunopositive area within a predetermined region. Briefly, for the Pcna/Sox2 double labeling, the area of Sox2-positive cells was outlined and measured, followed by manual thresholding and analysis of the Pcna-immunopositive area within this region. For the Ho-1, L-plastin, and vimentin stainings, a fixed area within the central retina (~0.04 mm^2^, covering all neuroretinal layers, with the GCL excluded for quantification of the Ho-1 and vimentin stainings as Müller glia endfeet would interfere with the analysis) or tectum (0.10 mm^2^ for the vimentin staining, with the PGZ excluded from the analysis since we were most interested in quantifying expression in the glial fibers, or 0.15 mm^2^ for the L-plastin staining) was first delineated. Next, the area occupied by L-plastin-positive cells or vimentin-positive signal within this region was defined via automated thresholding (Default, RenyiEntropy and Otsu for Ho-1, L-plastin, and vimentin, respectively), and divided by that region. Finally, for the γH2AX staining, the immunopositive signal was determined within one specific retinal and tectal nuclear layer via manual thresholding, i.e., in a predefined area in the INL (~0.02 mm^2^) and the PGZ (~0.04 mm^2^) for the retina and the optic tectum, respectively. Quantification was performed on at minimum three mid-sagittal or central coronal sections per fish and then averaged. Three to five fish were analyzed per age group.

**Table 2:** List of the primary antibodies used for immunohistochemistry.

Antibody	Manufacturer	Catalog number	Host species	Monoclonal or polyclonal	Dilution
BrdU	Abcam	Ab6326	Rat	Monoclonal	1:400
Ho-1	Enzo Life Sciences	ADI-SPA-896	Rabbit	Polyclonal	1:100
L-plastin	Genetex	GTX124420	Rabbit	Polyclonal	1:200
Pcna	Abcam	Ab29	Mouse	Monoclonal	1:500
Sox2	Sigma-Aldrich	SAB2701800	Rabbit	Polyclonal	1:1000
TH	Merck-Millipore	AB152	Rabbit	Polyclonal	1:400
Vimentin	Sigma-Aldrich	V5255	Mouse	Monoclonal	1:400
γH2AX	Genetex	GTX127342	Rabbit	Polyclonal	1:200

### Western blotting

For quantitative analysis of 4-HNE, fish of the four age groups were first euthanized in 0.1% Tris-buffered tricaine. Next, retinas as well as optic tecti (both left and right) were dissected, pooled per fish, and homogenized in lysis buffer containing 10 mM Tris-HCl pH 8, 1% Triton X-100, 150 mM NaCl, 0.1% SDS, 0.5% sodium deoxycholate, 0.2% sodium azide, and protease inhibitors (Roche). Homogenates were loaded at 20 µg onto 4–12% Bis-Tris gels (Bio-Rad), and then transferred onto nitrocellulose membranes (Bio-Rad). Mouse monoclonal anti-4-HNE (1:100; ab48506; Abcam) primary antibody was incubated overnight at 4 °C, followed by 45 min incubation with goat-anti-mouse horseradish-conjugated secondary antibody (1:5000; Bio-Rad). Protein bands were visualized using a luminol-based enhanced chemiluminescent kit (Bio-Rad) and an imaging system (Bio-Rad; ChemiDoc MP imaging system). Next, bands with molecular weights between 40 and 110 kDa were quantified by densitometry (Image Lab 4.1; Bio-Rad). As a loading control and for normalization of protein values, Swift membrane total protein staining (G-Biosciences) was used. Per age group, three fish were analyzed. All data are shown as relative to the young adult fish (6 weeks), which was set as 100%.

### Optokinetic response test

Visual acuity was investigated in 6-week-, 12-week-, 18-week-, and 24-week-old killifish using a virtual-reality chamber (OptoMotry; Cerebral Mechanics; Medicine Hat; AB; Canada). Briefly, fish were anesthetized for a short time in 0.03% Tris-buffered tricaine. Next, they were positioned in a custom-made glass chamber in which a continuous water flow was provided, allowing them to reawaken and breathe while being immobilized. The glass chamber was then placed in the middle of a virtual cylinder, displaying vertical sine wave gratings projected onto four computer monitors. Real-time video feedback was provided via a camera placed above the glass chamber. For each fish, eye movements were recorded and the maximal spatial frequency was determined, keeping velocity and contrast fixed at 15°/s and 100%, respectively. Each trial started with a spatial frequency of 0.02 (c/d), which increased via a staircase procedure. Fish were allowed to adapt to the system one day on beforehand. For every age group, visual acuity of at least 20 fish was assessed.

### Statistical analysis

For all statistical tests and creation of representative graphs, GraphPad Prism 8 software was used. Normal distribution was tested using the Kolmogorov–Smirnov normality test, and equality of variances was determined via the Brown–Forsythe test, and these assumptions were met in all cases. Significant differences between age groups were evaluated using one- or two-way ANOVA. Data are represented as mean ± standard error of mean and the exact number of biologically independent samples within each group (*n*) is indicated in the graphs. For all statistical tests, *p* < 0.05 was considered significantly different.

### Reporting summary

Further information on research design is available in the Nature Research Reporting Summary linked to this article.

## Supplementary information

Supplementary Information

Reporting Summary

## Data Availability

The data sets generated and/or analyzed during the current study are available from the corresponding author upon reasonable request.

## References

[1] Department of Economic and Social Affairs of the United Nations. *World Population Ageing 2019* (Econ. Soc. Aff., Popul. Division, 2019).

[2] World Health Organisation. *Global Action Plan on the Public Health Response to Dementia 2017–2025* (World Health Org., 2017).

[3] Cao Q (2020). The prevalence of dementia: a systematic review and meta-analysis. J. Alzheimers Dis..

[4] Vanhunsel S, Beckers A, Moons L (2020). Designing neuroreparative strategies using aged regenerating animal models. Ageing Res. Rev..

[5] Chader GJ, Taylor A (2013). Preface: the aging eye: normal changes, age-related diseases, and sight-saving approaches. Investig. Ophthalmol. Vis. Sci..

[6] Salvi SM, Akhtar S, Currie Z (2006). Ageing changes in the eye. Postgrad. Med. J..

[7] López-Otín C, Blasco MA, Partridge L, Serrano M, Kroemer G (2013). The hallmarks of aging. Cell.

[8] Mattson MP, Arumugam TV (2018). Hallmarks of brain aging: adaptive and pathological modification by metabolic states. Cell Metab..

[9] Chinta SJ (2015). Cellular senescence and the aging brain. Exp. Gerontol..

[10] Cellerino A, Valenzano DR, Reichard M (2016). From the bush to the bench: the annual *Nothobranchius* fishes as a new model system in biology. Biol. Rev..

[11] Kim Y, Nam HG, Valenzano DR (2016). The short-lived African turquoise killifish: an emerging experimental model for ageing. Dis. Model. Mech..

[12] Van houcke, J. et al. Modeling neuroregeneration and neurorepair in an aging context: the power of a teleost model. *Front. Cell Dev. Biol.* **9**, 619197 (2021). 10.3389/fcell.2021.619197 PMC8012675 33816468

[13] Genade T (2005). Annual fishes of the genus *Nothobarnchius* as a model system for aging research. Aging Cell.

[14] Terzibasi E (2008). Large differences in aging phenotype between strains of the short-lived annual fish *Nothobranchius furzeri*. PLoS ONE.

[15] Valenzano DR, Terzibasi E, Cattaneo A, Domenici L, Cellerino A (2006). Temperature affects longevity and age-related locomotor and cognitive decay in the short-lived fish: *Nothobranchius furzeri*. Aging Cell.

[16] Tozzini ET, Baumgart M, Battistoni G, Cellerino A (2012). Adult neurogenesis in the short-lived teleost *Nothobranchius furzeri*: localization of neurogenic niches, molecular characterization and effects of aging. Aging Cell.

[17] Baumgart M (2014). RNA-seq of the aging brain in the short-lived fish *N. furzeri*—conserved pathways and novel genes associated with neurogenesis. Aging Cell.

[18] Hartmann N (2011). Mitochondrial DNA copy number and function decrease with age in the short-lived fish *Nothobranchius furzeri*. Aging Cell.

[19] Reichwald K (2015). Insights into sex chromosome evolution and aging from the genome of a short-lived fish. Cell.

[20] Valenzano DR (2015). The African turquoise killifish genome provides insights into evolution and genetic architecture of lifespan. Cell.

[21] Polačik M, Blažek R, Reichard M (2016). Laboratory breeding of the short-lived annual killifish *Nothobranchius furzeri*. Nat. Protoc..

[22] Bollaerts I (2018). Complementary research models and methods to study axonal regeneration in the vertebrate retinofugal system. Brain Struct. Funct..

[23] Mirzaei N (2020). Alzheimer’s retinopathy: seeing disease in the eyes. Front. Neurosci..

[24] Guo L, Normando EM, Shah PA, De Groef L, Cordeiro MF (2018). Oculo-visual abnormalities in Parkinson’s disease: possible value as biomarkers. Mov. Disord..

[25] Vandenabeele M (2021). The App NL-G-F mouse retina is a site for preclinical Alzheimer’s disease diagnosis and research. Acta Neuropathol. Commun..

[26] Veys L (2019). Retinal α-synuclein deposits in Parkinson’s disease patients and animal models. Acta Neuropathol..

[27] Cerveny KL, Varga M, Wilson SW (2012). Continued growth and circuit building in the anamniote visual system. Dev. Neurobiol..

[28] Tsai SB (2007). Differential effects of genotoxic stress on both concurrent body growth and gradual senescence in the adult zebrafish. Aging Cell.

[29] Van houcke J (2019). Extensive growth is followed by neurodegenerative pathology in the continuously expanding adult zebrafish retina. Biogerontology.

[30] Gilmer LK, Ansari MA, Roberts KN, Scheff SW (2010). Age-related mitochondrial changes after traumatic brain injury. J. Neurotrauma.

[31] Shao CX, Roberts KN, Markesbery WR, Scheff SW, Lovell MA (2006). Oxidative stress in head trauma in aging. Free Radic. Biol. Med..

[32] Zhang B, Bailey WM, McVicar AL, Gensel JC (2016). Age increases reactive oxygen species production in macrophages and potentiates oxidative damage after spinal cord injury. Neurobiol. Aging.

[33] Poon HF, Calabrese V, Scapagnini G, Butterfield DA (2004). Free radicals: key to brain aging and heme oxygenase as a cellular response to oxidative stress. J. Gerontol. - Ser. A Biol. Sci. Med. Sci..

[34] Rosa P (2018). Heme oxygenase-1 and brain oxysterols metabolism are linked to Egr-1 expression in aged mice cortex, but not in hippocampus. Front. Aging Neurosci..

[35] Salminen A (2011). Astrocytes in the aging brain express characteristics of senescence-associated secretory phenotype. Eur. J. Neurosci..

[36] Telegina DV, Kozhevnikova OS, Kolosova NG (2018). Changes in retinal glial cells with age and during development of age-related macular degeneration. Biochemistry.

[37] Jurisch-Yaksi N, Yaksi E, Kizil C (2020). Radial glia in the zebrafish brain: functional, structural, and physiological comparison with the mammalian glia. Glia.

[38] Damani MR (2011). Age-related alterations in the dynamic behavior of microglia. Aging Cell.

[39] Koellhoffer EC, McCullough LD, Ritzel RM (2017). Old maids: aging and its impact on microglia function. Int. J. Mol. Sci..

[40] Streit WJ, Xue QS (2010). The brain’s aging immune system. Aging Dis..

[41] Von Bernhardi R, Tichauer JE, Eugenín J (2010). Aging-dependent changes of microglial cells and their relevance for neurodegenerative disorders. J. Neurochem..

[42] Erickson, M. A. & Banks, W. A. Age-associated changes in the immune system and blood–brain barrier functions. *Int. J. Mol. Sci.* **20**, 1632 (2019). 10.3390/ijms20071632 PMC6479894 30986918

[43] Apple DM, Kokovay E (2017). Vascular niche contribution to age-associated neural stem cell dysfunction. Am. J. Physiol. Circ. Physiol..

[44] Capilla-Gonzalez V, Cebrian-Silla A, Guerrero-Cazares H, Garcia-Verdugo JM, Quiñones-Hinojosa A (2014). Age-related changes in astrocytic and ependymal cells of the subventricular zone. Glia.

[45] Decarolis NA, Kirby ED, Wyss-Coray T, Palmer TD (2015). The role of the microenvironmental niche in declining stem-cell functions associated with biological aging. Cold Spring Harb. Perspect. Med..

[46] Lee SW, Clemenson GD, Gage FH (2012). New neurons in an aged brain. Behav. Brain Res..

[47] Smith LK, White CW, Villeda SA (2018). The systemic environment: at the interface of aging and adult neurogenesis. Cell Tissue Res..

[48] Matsui H, Kenmochi N, Namikawa K (2019). Age- and α-synuclein-dependent degeneration of dopamine and noradrenaline neurons in the annual killifish *Nothobranchius furzeri*. Cell Rep..

[49] Ramirez AI (2017). The role of microglia in retinal neurodegeneration: Alzheimer’s disease, Parkinson, and glaucoma. Front. Aging Neurosci..

[50] Martins, R. R., Zamzam, M., Moosajee, M., Thummel, R. & MacDonald, R. B. Age-related degeneration leads to gliosis but not regeneration in the zebrafish retina. (2020). 10.1101/2020.06.28.174821.

[51] Weale RA (1975). Senile changes in visual acuity. Trans. Ophthalmol. Soc. UK.

[52] Arden GB, Jacobson JJ (1978). A simple grating test for contrast sensitivity: preliminary results indicate value in screening for glaucoma. Investig. Ophthalmol. Vis. Sci..

[53] Skalka HW (1980). Effect of age on Arden grating acuity. Br. J. Ophthalmol..

[54] Marshall J (1987). The ageing retina: physiology or pathology. Eye.

[55] Al-Ubaidi MR, Naash MI, Conley SM (2013). A perspective on the role of the extracellular matrix in progressive retinal degenerative disorders. Investig. Ophthalmol. Vis. Sci..

[56] Medeiros ADM, Silva RH (2019). Sex differences in Alzheimer’s disease: where do we stand?. J. Alzheimer’s Dis..

[57] Hanamsagar R, Bilbo SD (2016). Sex differences in neurodevelopmental and neurodegenerative disorders: focus on microglial function and neuroinflammation during development. J. Steroid Biochem. Mol. Biol..

[58] Arslan-Ergul A, Adams MM (2014). Gene expression changes in aging Zebrafish (Danio rerio) brains are sexually dimorphic. BMC Neurosci..

[59] Ampatzis K, Makantasi P, Dermon CR (2012). Cell proliferation pattern in adult zebrafish forebrain is sexually dimorphic. Neuroscience.

[60] Kerr N, Dietrich DW, Bramlett HM, Raval AP (2019). Sexually dimorphic microglia and ischemic stroke. CNS Neurosci. Ther..

[61] Hernandez-Segura A, Nehme J, Demaria M (2018). Hallmarks of cellular senescence. Trends Cell Biol..

[62] Kishi S (2003). The zebrafish as a vertebrate model of functional aging and very gradual senescence. Exp. Gerontol..

[63] Hsu CY, Chiu YC, Hsu WL, Chan YP (2008). Age-related markers assayed at different developmental stages of the annual fish *Nothobranchius rachovii*. J. Gerontol. - Ser. A Biol. Sci. Med. Sci..

[64] Liu C (2012). Differential expression of aging biomarkers at different life stages of the annual fish *Nothobranchius guentheri*. Biogerontology.

[65] Terzibasi E, Valenzano DR, Cellerino A (2007). The short-lived fish *Nothobranchius furzeri* as a new model system for aging studies. Exp. Gerontol..

[66] Ding, L., Kuhne, W. W., Hinton, D. E., Song, J. & Dynan, W. S. Quantifiable biomarkers of normal aging in the Japanese Medaka fish (Oryzias latipes). *PLoS ONE* **5**, e13287 (2010). 10.1371/journal.pone.0013287 PMC2952620 20949019

[67] Arslan-Ergul A, Erbaba B, Karoglu ET, Halim DO, Adams MM (2016). Short-term dietary restriction in old zebrafish changes cell senescence mechanisms. Neuroscience.

[68] Jurk D (2012). Postmitotic neurons develop a p21-dependent senescence-like phenotype driven by a DNA damage response. Aging Cell.

[69] Tan FCC, Hutchison ER, Eitan E, Mattson MP (2014). Are there roles for brain cell senescence in aging and neurodegenerative disorders?. Biogerontology.

[70] Van Deursen JM (2014). The role of senescent cells in ageing. Nature.

[71] Van houcke J, De Groef L, Dekeyster E, Moons L (2015). The zebrafish as a gerontology model in nervous system aging, disease, and repair. Ageing Res. Rev..

[72] Franceschi C (2007). Inflammaging and anti-inflammaging: a systemic perspective on aging and longevity emerged from studies in humans. Mech. Ageing Dev..

[73] Van houcke, J. et al. Aging impairs the essential contributions of non-glial progenitors to neurorepair in the dorsal telencephalon of the Killifish *N. furzeri*. (2021). 10.1101/2021.02.26.433041. 10.1111/acel.13464 PMC8441397 34428340

[74] Van houcke J (2017). Successful optic nerve regeneration in the senescent zebrafish despite age-related decline of cell intrinsic and extrinsic response processes. Neurobiol. Aging.

[75] Petzold A (2013). The transcript catalogue of the short-lived fish *Nothobranchius furzeri* provides insights into age-dependent changes of mRNA levels. BMC Genom..

[76] Hu WT (2019). CSF cytokines in aging, multiple sclerosis, and dementia. Front. Immunol..

[77] Lana, D., Ugolini, F., Nosi, D., Wenk, G. L. & Giovannini, M. G. The emerging role of the interplay among astrocytes, microglia, and neurons in the hippocampus in health and disease. *Front. Aging Neurosci.* **13**, 651973 (2021). 10.3389/fnagi.2021.651973 PMC8055856 33889084

[78] Mészáros Á (2020). Neurovascular inflammaging in health and disease. Cells.

[79] Angelova DM, Brown DR (2019). Microglia and the aging brain: are senescent microglia the key to neurodegeneration?. J. Neurochem..

[80] Sandhir R, Puri V, Klein RM, Berman NEJ (2004). Differential expression of cytokines and chemokines during secondary neuron death following brain injury in old and young mice. Neurosci. Lett..

[81] Rea IM (2018). Age and age-related diseases: role of inflammation triggers and cytokines. Front. Immunol..

[82] Zhang B, Bailey WM, Braun KJ, Gensel JC (2015). Age decreases macrophage IL-10 expression: implications for functional recovery and tissue repair in spinal cord injury. Exp. Neurol..

[83] Hardeland R (2018). Brain inflammaging: roles of melatonin, circadian clocks and sirtuins. J. Clin. Cell. Immunol..

[84] D’Angelo S, Mele E, Di Filippo F, Viggiano A, Meccariello R (2021). Sirt1 activity in the brain: simultaneous effects on energy homeostasis and reproduction. Int. J. Environ. Res. Public Health.

[85] Jiao, F. & Gong, Z. The beneficial roles of SIRT1 in neuroinflammation-related diseases. *Oxid. Med. Cell. Longev.* **2020**, 6782872 (2020). 10.1155/2020/6782872 PMC7519200 33014276

[86] Sarubbo, F., Tejada, S., Esteban, S., Jimenez-García, M. & Moranta, D. Resveratrol, SIRT1, oxidative stress, and brain aging. *Aging*, 319–326 (2020). 10.1016/b978-0-12-818698-5.00033-x.

[87] Duan W (2013). Sirtuins: from metabolic regulation to brain aging. Front. Aging Neurosci..

[88] Kim DH, Jung IH, Kim DH, Park SW (2019). Knockout of longevity gene Sirt1 in zebrafish leads to oxidative injury, chronic inflammation, and reduced life span. PLoS ONE.

[89] Sheng W (2018). Effect of resveratrol on sirtuins, OPA1, and Fis1 expression in adult zebrafish retina. Investig. Ophthalmol. Vis. Sci..

[90] Bourgognon J-M, Cavanagh J (2020). The role of cytokines in modulating learning and memory and brain plasticity. Brain Neurosci. Adv..

[91] Xie Z, Morgan TE, Rozovsky I, Finch CE (2003). Aging and glial responses to lipopolysaccharide in vitro: greater induction of IL-1 and IL-6, but smaller induction of neurotoxicity. Exp. Neurol..

[92] Ogai K (2014). Upregulation of leukemia inhibitory factor (LIF) during the early stage of optic nerve regeneration in zebrafish. PLoS ONE.

[93] Van Dyck A (2021). Müller glia–myeloid cell crosstalk accelerates optic nerve regeneration in the adult zebrafish. Glia.

[94] Maher FO, Martin DSD, Lynch MA (2004). Increased IL-1β in cortex of aged rats is accompanied by downregulation of ERK and PI-3 kinase. Neurobiol. Aging.

[95] Gee JR, Ding Q, Keller JN (2006). Age-related alterations of apolipoprotein E and interleukin-1β in the aging brain. Biogerontology.

[96] Gayle D (1999). Basal and IL-1β-stimulated cytokine and neuropeptide mRNA expression in brain regions of young and old Long-Evans rats. Mol. Brain Res..

[97] Liddelow SA (2017). Neurotoxic reactive astrocytes are induced by activated microglia. Nature.

[98] Edelmann K (2013). Increased radial glia quiescence, decreased reactivation upon injury and unaltered neuroblast behavior underlie decreased neurogenesis in the aging zebrafish telencephalon. J. Comp. Neurol..

[99] Ahlenius H, Visan V, Kokaia M, Lindvall O, Kokaia Z (2009). Neural stem and progenitor cells retain their potential for proliferation and differentiation into functional neurons despite lower number in aged brain. J. Neurosci..

[100] Kishi S (2008). The identification of zebrafish mutants showing alterations in senescence-associated biomarkers. PLoS Genet..

[101] Kishi S, Slack BE, Uchiyama J, Zhdanova IV (2009). Zebrafish as a genetic model in biological and behavioral gerontology: where development meets aging in vertebrates—a mini-review. Gerontology.

[102] Fu J, Nagashima M, Guo C, Raymond PA, Wei X (2018). Novel animal model of crumbs-dependent progressive retinal degeneration that targets specific cone subtypes. Investig. Ophthalmol. Vis. Sci..

[103] Raymond PA, Easter SS (1983). Postembryonic growth of the optic tectum in goldfish. I. Location of germinal cells and numbers of neurons produced. J. Neurosci..

[104] Valenzano DR (2006). Resveratrol prolongs lifespan and retards the onset of age-related markers in a short-lived vertebrate. Curr. Biol..

[105] Gorsuch RA (2017). Sox2 regulates Müller glia reprogramming and proliferation in the regenerating zebrafish retina via Lin28 and Ascl1a. Exp. Eye Res..

[106] Takeuchi A, Okubo K (2013). Post-proliferative immature radial glial cells female-specifically express aromatase in the medaka optic tectum. PLoS ONE.

[107] Deoliveira-mello L, Lara JM, Arevalo R, Velasco A, Mack AF (2019). Sox2 expression in the visual system of two teleost species. Brain Res..

