# Supplementary material for: The killifish visual system as an in vivo model to study brain aging and rejuvenation
Source: NPJ Aging Mech Dis. 2021 Aug 17;7:22. doi: 10.1038/s41514-021-00077-4 (PMC8371010; doi:10.1038/s41514-021-00077-4)
Supplement: Supplementary file 1 — Supplementary Information [file 41514_2021_77_MOESM1_ESM.pdf]

SUPPLEMENTARY FIGURES

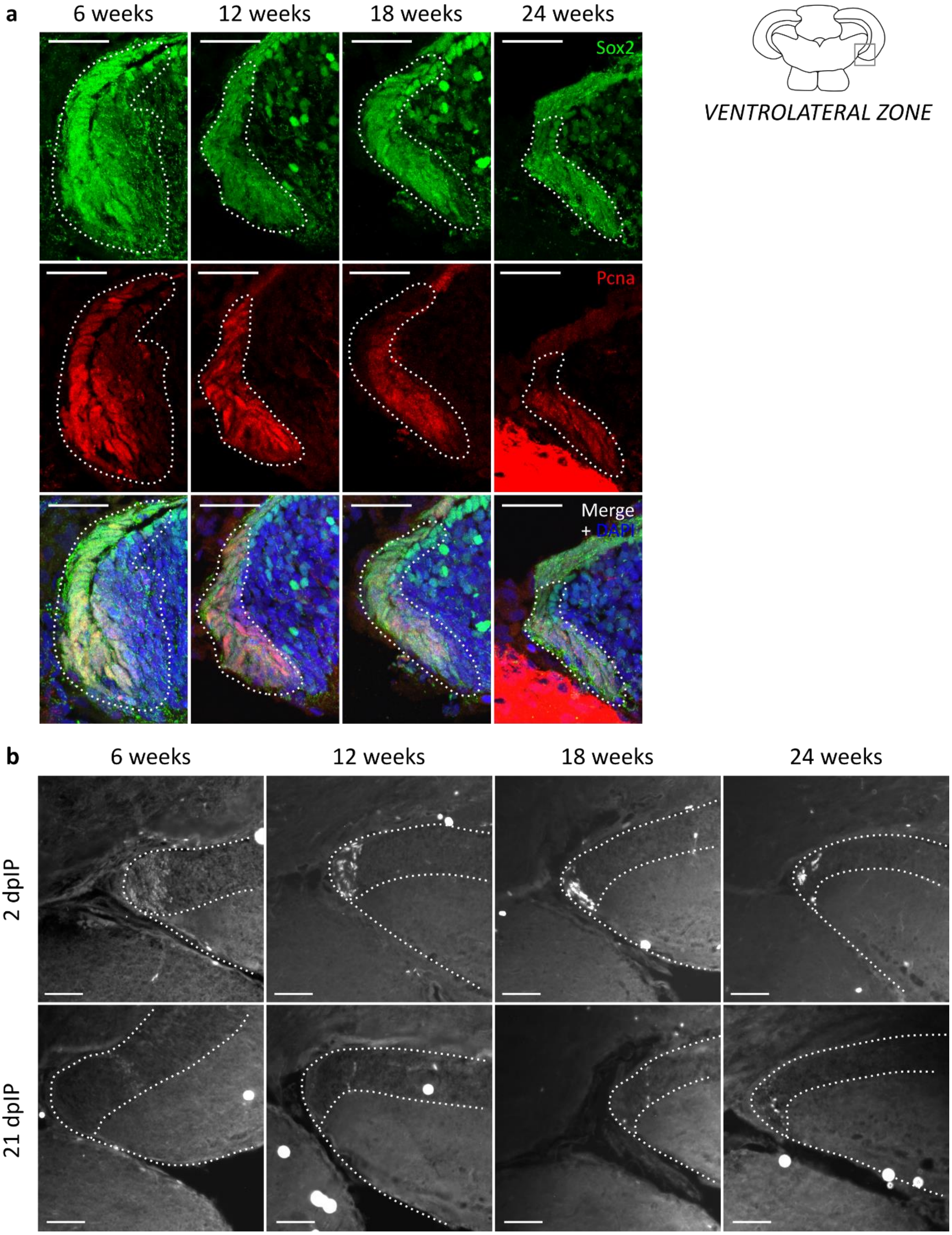

Supplementary Fig. 1 Decreased number of (proliferating) stem cells and reduced neurogenic ability in the ventrolateral mitotic zone of the aged killifish optic tectum. a Representative images of Pcna- and Sox2 double-stained tectal sections of fish of all four age groups, visualizing an age-associated reduction in the number of neural stem/progenitor cells (NSPCs) (Sox2-positive) as well as the number of proliferating NSPCs (Sox2 and Pcna double-positive) in the ventrolateral zone of the optic tectum.

Scale bar = 25 µm. **b** Microscopic images of tectal sections containing the ventrolateral neurogenic zone immunostained for BrdU disclose that at 2 dpIP of BrdU, a clear reduction in the number of BrdU-positive cells is observed upon aging. Following a 21 days chase, the number of BrdU-positive cells as well as the distance from the mitotic niche to the furthest BrdU-positive cell, are both reduced in older fish. Scale bar = 50 µm. Abbreviations: DAPI, 4′,6-diamidino-2-phenylindole; Sox2, SRY-Box Transcription Factor 2; Pcna, Proliferating cell nuclear antigen; dpIP, days *post* intraperitoneal injection.

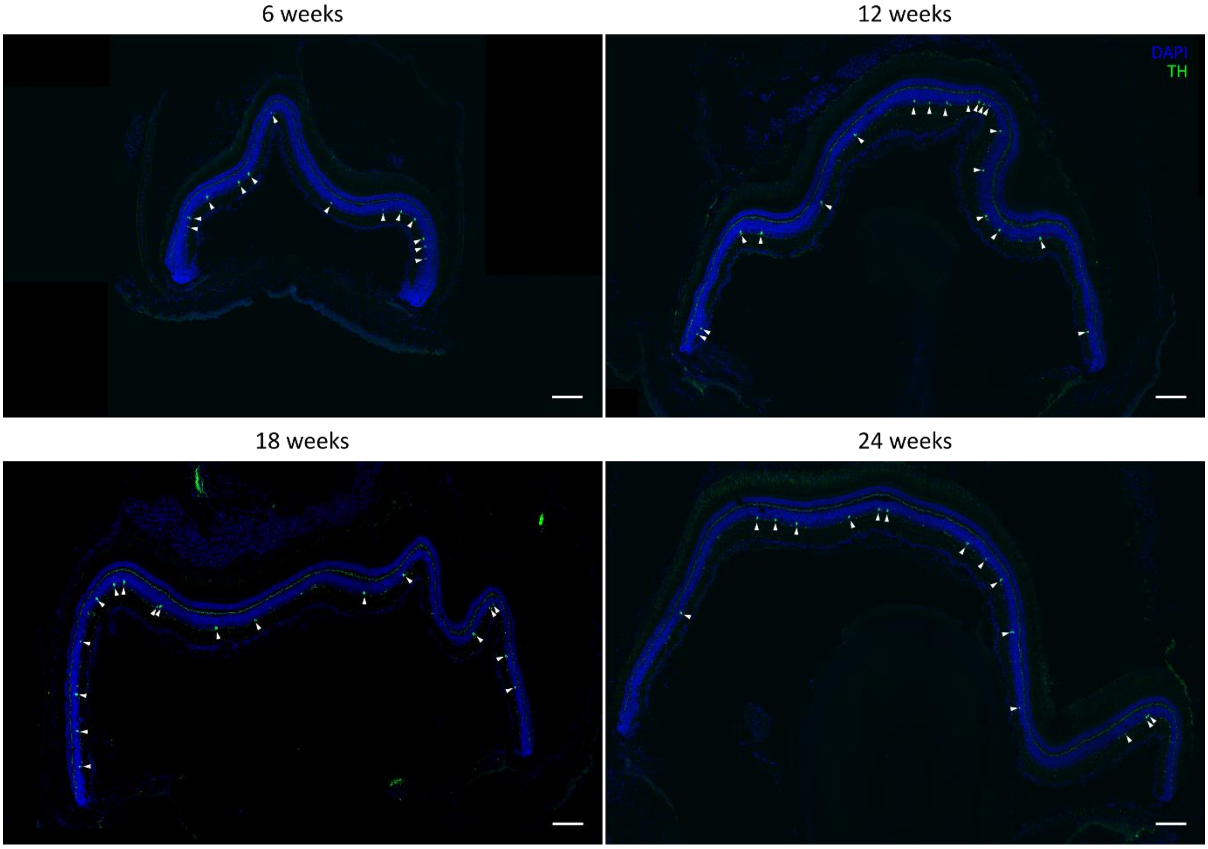

Supplementary Fig. 2 Decreased absolute number of dopaminergic amacrine cells in the retina of the aged killifish. Representative pictures of midsagittal retinal cryosections immunolabeled for TH demonstrate a visible decrease in the number of TH-expressing (displaced) amacrine cells between the age of 12 and 18w old fish (white arrowheads). Scale bar = 100 µm. Abbreviations: DAPI, 4′,6-diamidino-2-phenylindole; TH, tyrosine hydroxylase.

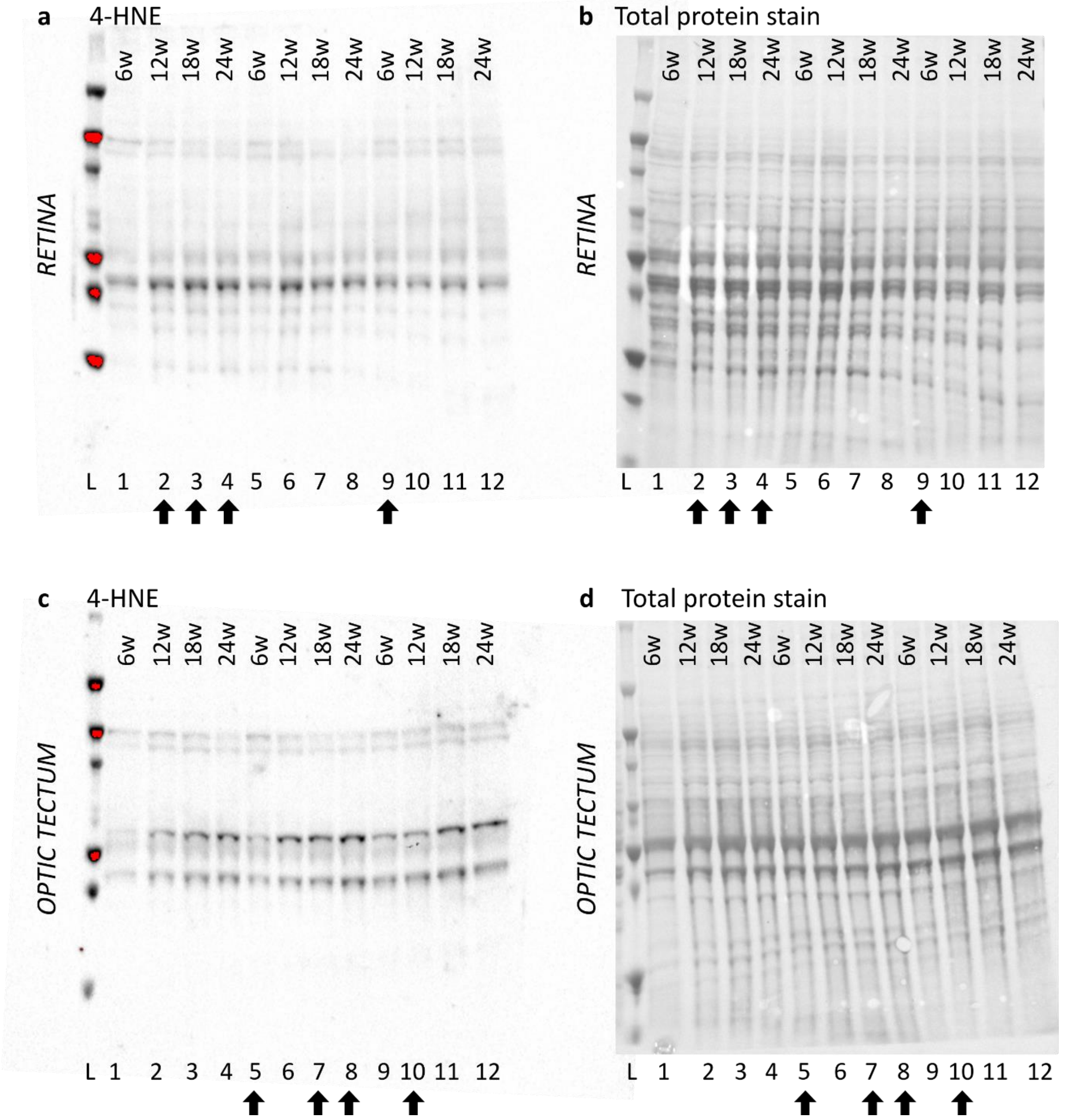

Supplementary Fig. 3 Full-length images of the Western blots for 4-HNE. a Western blot of 4-HNE levels in the retina of killifish of different ages. **b** Total protein stain on blot with killifish retinal samples. **c** Western blot of 4-HNE levels in the optic tectum of killifish of different ages. **d** Total protein stain on blot with samples of the killifish optic tectum. For every blot: lanes 1, 5, 9 contain samples of 6w old killifish; lanes 2, 6, 10 of 12w old killifish; lanes 3, 7, 11 of 18w old killifish; and lanes 4, 8, 12 of 24w old killifish; with samples of one fish per lane. Arrows highlight lanes used in figure 2a. Abbreviations: 4-HNE, 4-hydroxynonenal; L, protein ladder; w, weeks.
