# Supplementary material for: The killifish visual system as an in vivo model to study brain aging and rejuvenation
Source: NPJ Aging Mech Dis. 2021 Aug 17;7:22. doi: 10.1038/s41514-021-00077-4 (PMC8371010; doi:10.1038/s41514-021-00077-4)
Supplement: Supplementary file 2 — Reporting Summary [file 41514_2021_77_MOESM2_ESM.pdf]

nature portfolio

Corresponding author(s): Lieve Moons

Last updated by author(s): Jul 9, 2021

Reporting Summary

Nature Portfolio wishes to improve the reproducibility of the work that we publish. This form provides structure for consistency and transparency in reporting. For further information on Nature Portfolio policies, see our Editorial Policies and the Editorial Policy Checklist.

Statistics

For all statistical analyses, confirm that the following items are present in the figure legend, table legend, main text, or Methods section.

n/a	Confirmed	
☐	☒	The exact sample size (*n*) for each experimental group/condition, given as a discrete number and unit of measurement
☐	☒	A statement on whether measurements were taken from distinct samples or whether the same sample was measured repeatedly
☐	☒	The statistical test(s) used AND whether they are one- or two-sided<br>*Only common tests should be described solely by name; describe more complex techniques in the Methods section.*
☒	☐	A description of all covariates tested
☐	☒	A description of any assumptions or corrections, such as tests of normality and adjustment for multiple comparisons
☐	☒	A full description of the statistical parameters including central tendency (e.g. means) or other basic estimates (e.g. regression coefficient) AND variation (e.g. standard deviation) or associated estimates of uncertainty (e.g. confidence intervals)
☒	☐	For null hypothesis testing, the test statistic (e.g. *F*, *t*, *r*) with confidence intervals, effect sizes, degrees of freedom and *P* value noted<br>*Give P values as exact values whenever suitable.*
☒	☐	For Bayesian analysis, information on the choice of priors and Markov chain Monte Carlo settings
☒	☐	For hierarchical and complex designs, identification of the appropriate level for tests and full reporting of outcomes
☒	☐	Estimates of effect sizes (e.g. Cohen's *d*, Pearson's *r*), indicating how they were calculated

Our web collection on statistics for biologists contains articles on many of the points above.

Software and code

Policy information about availability of computer code

Data collection	No software was used for data collection.
Data analysis	Quantification of data was performed using FIJI (ImageJ). For all statistical tests, GraphPad Prism 8 software was used.

For manuscripts utilizing custom algorithms or software that are central to the research but not yet described in published literature, software must be made available to editors and reviewers. We strongly encourage code deposition in a community repository (e.g. GitHub). See the Nature Portfolio guidelines for submitting code & software for further information.

Data

Policy information about availability of data

All manuscripts must include a data availability statement. This statement should provide the following information, where applicable:

- Accession codes, unique identifiers, or web links for publicly available datasets
- A description of any restrictions on data availability
- For clinical datasets or third party data, please ensure that the statement adheres to our policy

The data sets generated and/or analyzed during the current study are available from the corresponding author upon reasonable request.

Field-specific reporting

Please select the one below that is the best fit for your research. If you are not sure, read the appropriate sections before making your selection.

☒ Life sciences ☐ Behavioural & social sciences ☐ Ecological, evolutionary & environmental sciences

For a reference copy of the document with all sections, see nature.com/documents/nr-reporting-summary-flat.pdf

Life sciences study design

All studies must disclose on these points even when the disclosure is negative.

Sample size	Sample size was determined based on literature, in-house experience of similar experiments performed in zebrafish and pilot experiments that determined biological variability.
Data exclusions	Every condition was tested for outliers using GraphPad Prism 8 (ROUT method, Q = 1%). In case of outliers, these data were excluded.
Replication	For all experiments, biological replicates were used within one age group, indicated in the graphs with n. During optimization, young and old killifish were tested. These results were not included in the presented data but do confirm reproducibility in house.
Randomization	For every age group, fish from different aquaria were allocated to the various experimental readouts.
Blinding	All data analysis were performed blindly, except for the optokinetic response test as phenotypical characteristics clearly indicated the age of the fish.

Reporting for specific materials, systems and methods

We require information from authors about some types of materials, experimental systems and methods used in many studies. Here, indicate whether each material, system or method listed is relevant to your study. If you are not sure if a list item applies to your research, read the appropriate section before selecting a response.

Materials & experimental systems

n/a	Involved in the study	
☐	☒	Antibodies
☒	☐	Eukaryotic cell lines
☒	☐	Palaeontology and archaeology
☐	☒	Animals and other organisms
☒	☐	Human research participants
☒	☐	Clinical data
☒	☐	Dual use research of concern

Methods

n/a	Involved in the study	
☒	☐	ChIP-seq
☒	☐	Flow cytometry
☒	☐	MRI-based neuroimaging

Antibodies

Antibodies used	Monoclonal rat anti-BrdU (Abcam; Ab6326; 1:400)<br>Polyclonal rabbit anti-Ho-1 (Enzo Life Sciences; ADI-SPA-896; 1:100)<br>Polyclonal rabbit anti-L-plastin (Genetex; GTX124420; 1:200)<br>Monoclonal mouse anti-Pcna (Abcam; Ab29; 1:500)<br>Polyclonal rabbit anti-Sox2 (Sigma-Aldrich; SAB2701800; 1:1000)<br>Polyclonal rabbit anti-TH (Merck-Millipore; AB152; 1:400)<br>Monoclonal mouse anti-vimentin (Sigma-Aldrich; V5255; 1:400)<br>Polyclonal rabbit anti-γH2AX (Genetex; GTX127342; 1:200)<br>Monoclonal mouse anti-4HNE (Abcam; ab48506; 1:100)
Validation	Using a novel model organism, only few antibodies have been described to work in killifish (for example anti-TH by Matsui et al. 2019, anti-BrdU by Coolen et al. 2020). Specificity was determined based on literature using zebrafish and medaka as model organisms. During optimization, negative and positive controls (for example zebrafish tissues) were taken along.

Animals and other organisms

Policy information about studies involving animals; ARRIVE guidelines recommended for reporting animal research

Laboratory animals	Nothobranchius furzeri, GRZ-AD strain, female.
Wild animals	The study did not involve wild animals.

Field-collected samples	The study did not involve samples collected from the field.
Ethics oversight	All experiments were approved by the institutional Animal Ethics Committee of the KU Leuven and strictly followed the European Communities Council Directive of 20 October 2010 (2010/63/EU).

Note that full information on the approval of the study protocol must also be provided in the manuscript.
